# A systemically deliverable Vaccinia virus with increased capacity for intertumoral and intratumoral spread effectively treats pancreatic cancer

**DOI:** 10.1136/jitc-2020-001624

**Published:** 2021-01-22

**Authors:** Giulia Marelli, Louisa S Chard Dunmall, Ming Yuan, Carmela Di Gioia, Jinxin Miao, Zhenguo Cheng, Zhongxian Zhang, Peng Liu, Jahangir Ahmed, Rathi Gangeswaran, Nicholas Lemoine, Yaohe Wang

**Affiliations:** 1 Centre for Cancer Biomarkers and Biotherapeutics, Barts Cancer Institute, Queen Mary University of London, London, UK; 2 National Centre for International Research in Cell and Gene Therapy, Zhengzhou University, Zhengzhou, Henan, China; 3 Academy of Chinese Medicine Science, Henan University of Chinese Medicine, Zhengzhou 450000, Henan Province, People’s Republic of China

**Keywords:** oncolytic viruses, immunomodulation, tumor microenvironment, macrophages, natural killer T-cells

## Abstract

**Background:**

Pancreatic cancer remains one of the most lethal cancers and is refractory to immunotherapeutic interventions. Oncolytic viruses are a promising new treatment option, but current platforms demonstrate limited efficacy, especially for inaccessible and metastatic cancers that require systemically deliverable therapies. We recently described an oncolytic vaccinia virus (VV), VVLΔTKΔN1L, which has potent antitumor activity, and a regime to enhance intravenous delivery of VV by pharmacological inhibition of pharmacological inhibition of PI3 Kinase δ (PI3Kδ) to prevent virus uptake by macrophages. While these platforms improve the clinical prospects of VV, antitumor efficacy must be improved.

**Methods:**

VVLΔTKΔN1L was modified to improve viral spread within and between tumors via viral B5R protein modification, which enhanced production of the extracellular enveloped virus form of VV. Antitumor immunity evoked by viral treatment was improved by arming the virus with interleukin-21, creating VVL-21. Efficacy, functional activity and synergy with α-programmed cell death protein 1 (α-PD1) were assessed after systemic delivery to murine and Syrian hamster models of pancreatic cancer.

**Results:**

VVL-21 could reach tumors after systemic delivery and demonstrated antitumor efficacy in subcutaneous, orthotopic and disseminated models of pancreatic cancer. The incorporation of modified B5R improved intratumoural accumulation of VV. VVL-21 treatment increased the numbers of effector CD8+ T cells within the tumor, increased circulating natural killer cells and was able to polarize macrophages to an M1 phenotype in vivo and in vitro. Importantly, treatment with VVL-21 sensitized tumors to the immune checkpoint inhibitor α-PD1.

**Conclusions:**

Intravenously administered VVL-21 successfully remodeled the suppressive tumor-microenvironment to promote antitumor immune responses and improve long-term survival in animal models of pancreatic cancer. Importantly, treatment with VVL-21 sensitized tumors to the immune checkpoint inhibitor α-PD1. Combination of PI3Kδ inhibition, VVL-21 and α-PD1 creates an effective platform for treatment of pancreatic cancer.

## Background

Cancer is a growing global burden, with incidence expected to increase by 62% worldwide by 2040, but traditional therapies are limited by poor efficacy and intolerable side effects.^1^ Pancreatic cancer (PaCa) is a particularly devastating example. It is the seventh leading cause of cancer death worldwide, with over 450 000 new cases and 432 000 deaths reported in 2018.^1^ While immunotherapeutics such as immune checkpoint inhibition (ICI) have emerged as a promising new approach for cancer treatment, PaCa in particular is unresponsive to ICI monotherapy.^2^ Therapeutics that have the ability to evoke immune activation within the tumor microenvironment (TME) are therefore sought to broaden the effectiveness of ICI in PaCa. A particular barrier to the effective treatment of PaCa is the inaccessibility of the tumor to treatment. Additionally, patients usually present with advanced disease that has metastasized to distant sites.

Oncolytic viral therapy (OVT) uses engineered viruses designed to selectively destroy cancer cells and activate antitumor immune responses. The ability of OVT to sensitize tumors to ICI therapy is being investigated and the combination of OVT and α-programmed cell death protein 1 (α-PD1) ICI treatment has shown increased response rates in melanoma compared with the use of either agent alone,^3^ suggesting that OVT can effectively modify the TME such that it becomes responsive to intervention with ICI. Vaccinia virus (VV) is a particularly strong candidate OVT for treatment of PaCa as it has a number of inherent features that render it superior to others in clinical development including, in particular, a lack of requirement for a specific surface receptor^4^; the ability to replicate in hypoxic environments that represent the treatment-resistant fractions of PaCa^5^; induction of immunogenic cell death pathways^6^ and an ability to induce vascular collapse within the TME.^7^ While delivery of most OVTs is limited to intratumoral injection, VV has been reported to reach tumors after intravenous delivery^8^ and systemic spread is enabled by the existence of multiple, antigenically distinct forms of the virus during the replication cycle, which allows evasion of the host immune system and infection of remote tumor sites.^9 10^ During its lifecycle, the virus produces two infectious forms and the extracellular enveloped virion (EEV) form is crucial for efficient cell-cell spread and long-range dissemination of the virus in vivo, as EEV can avoid clearance by the host immune response. However, most strains of VV only produce EEV at low levels (<1%).^11 12^

Despite these properties, many challenges need to be addressed to overcome the current limitations observed in clinical efficacy of VV, including a rational strain selection, informed gene modification to enhance safety and antitumor activity, enhancing the potential for systemic delivery, improving spread within and between tumors and selection of the most appropriate transgene(s) to compound the generation of antitumor immune responses.

We recently described a novel thymidine kinase (TK)-deleted, N1L gene-deleted VV, VVLΔTKΔN1L, which demonstrated potent gene-deletion-driven tumor specificity, activation of antitumor immunity and potent efficacy in a number of tumor models in vivo.^13–15^ Additionally, we have developed a platform to improve intravenous delivery of VV based on transient pharmacological inhibition of PI3 Kinase δ (PI3Kδ) to prevent uptake of the virus by macrophages, a major factor limiting systemic administration.^16^ This platform goes some way toward addressing the shortcomings of OVT identified above. However, in order to create a clinically valuable OVT, improvements in systemic spread and antitumor efficacy of VVLΔTKΔN1L are still required.

Here, we report a rational re-engineering of VVLΔTKΔN1L to enhance EEV production and spread of the virus within tumors. This was undertaken via incorporation of an additional copy of the signal peptide, stalk (S), transmembrane (T) and cytoplasmic tail (C) regions of the viral B5R gene, creating a new oncolytic VV (VVLΔTK-STCΔN1L) that was able to accumulate to higher levels within tumors on systemic delivery. We armed this virus with interleukin-21 (IL-21) to improve the induction of antitumor immune responses to create VVLΔTK-STCΔN1L-IL21 (referred to hereafter as VVL-21). IL-21 is a potent inducer of T cell activation in vivo^17^ and can inhibit the development of suppressive FOXP3 regulatory T (TReg) cells,^18^ induce maturation, activation and cytolytic potential of natural killer (NK) and NKT cells,^19 20^ promote B cell production of tumor-specific IgG^21^ and inhibit angiogenesis by reducing expression of VEGFR1 and TIE1 in endothelial cells.^22^ Significantly, there have been no reported adverse effects, even when administered at high doses,^23^ although the antitumor efficacy of IL-21 as a monotherapy appears limited in early clinical trials.^24^ VVL-21 demonstrated enhanced potential for systemic spread and had potent antitumor activity after intravenous and intraperitoneal delivery. Additionally, it was able to effectively sensitize PaCa to the ICI α-PD1.

## Materials and methods

### Viruses

VVΔTKΔN1L was described previously^25 26^ and tumor specificity ensured by rational deletion of TK and N1L genes.^13 14 16^ VVΔTKΔN1L-IL21 (containing the murine or human IL-21 cytokine) construction was described previously.^27^ For in vitro and mouse studies, VVL-21 armed with murine IL-21 was used. For hamster studies, VVL-21 armed with human IL-21 was used.

To construct the TK-B5R STC virus, the TK shuttle vector containing RFP flanked by LoxP sites described previously was used.^27^ The signal peptide of the viral B5R gene (SP) was amplified by PCR using forward primer (5’-*TTAATTAA*AAATAAAAATGAAAACGATTTCCG-3’) (*PacI* is underlined) and reverse primer (5’-*GCTAGCGAATTCAAGCTT*TGAATAAACAACAGC-3’) (*NheI*, *EcoRI* and *HindIII* are underlined). The B5R STC fragment (STALK+TM+CT) was amplified by PCR using forward primer (5’-*AAGCTT*TGTGTACGAACTAACGAAAAA-3’) (*HindIII* is underlined) and reverse primer (5’-*GCTAGC*TCACGGTAGCAATTTATGGAACT-3’) (*NheI* is underlined). The SP fragment was cloned into the pGEM-T easy vector (Promega) and designated pGEM-T easy-SP. STC fragment was cloned into *HindIII* and *NheI* sites of pGEM-T easy-SP to obtain pGEM-T easy-SP+STC. SP+STC was released from pGEM-T easy-SP+STC using *PacI* and *NheI* restriction enzymes and cloned into *PacI* and *NheI* sites of the TK-directed shuttle vector containing RFP flanked by LoxP sites as previously described.^27^ Viral construction and production were carried out as described previously.^15^ Of note, this method of purification results in intracellular mature virion (IMV) production. Improved EEV production only occurs during replication within tumor cells.

### In vivo studies

In subcutaneous tumor models, animals were assigned to treatment groups by matching tumor sizes prior to treatment. Tumor growth was measured using electronic calipers until tumors reached 1.44 cm^2^ (w×L) and the area plotted using the following formula:

Where w is width, L is length.

Tumor growth curves were terminated on the death of the first animal in each group, but group survival was monitored until the experimental end point and Kaplan-Meier survival plots generated.

DT6606 cells (3×10^6^ cells/mouse) were implanted subcutaneously into the right flanks of male C57Bl/6 mice aged 8 weeks. When the tumors were palpable (100 mm^3^), mice were stratified into treatment groups. Mice received 10 mg/kg CAL-101 or vehicle buffer via oral gavage 3 hours prior to virus (or phosphate-buffered saline (PBS)) injection at 1×10^8^ plaque-forming unit (PFU)/injection on days 0, 2 and 4 (one biological repeat was carried out) or at 1×10^8^ PFU/injection on days 0, 2, 4 and at 2×10^8^ PFU/injection on days 13, 15 and 17 (two biological repeats were carried out) as indicated and tumor growth measured twice a week. Viruses were resuspended in PBS injected intravenously via a tail vein. α-PD1 antibody was resuspended in PBS at final concentration of 200 µg/mouse and injected at days 2, 5 and 7 or 2, 5, 7, 15, 19 and 20 as indicated in the results. Mice were culled and cardiac puncture was performed to collect blood. Tumor and spleen were also collected and processed as detailed elsewhere in the online supplemental materials and methods. The samples were used for PCR, quantitative PCR, flow cytometry (FC), immunohistochemistry (IHC) staining or interferon (IFN)-γ ELISA assay. Note, for mouse studies, VVL-21 armed with a murine form of IL-21 was used.

10.1136/jitc-2020-001624.supp1 Supplementary data

### Orthotopic injection

Male C57Bl/6 mice aged 10 weeks were injected with 1×10^6^ DT6606 tumor cells into the tail of the pancreas. Mice were anesthetized and placed in the dorsal decubitus position, and a left subcostal incision made. The pancreas was carefully exposed, and 30 µL of tumor cell suspension were injected into the tail of the pancreas. A technically successful injection was characterized by the formation of a visible bubble within the pancreatic parenchyma. The needle was slowly withdrawn to avoid macroscopic cell spread from the injection site. The pancreas was then returned to the peritoneal cavity. Wounds were closed using surgical thread. Buprenorphine (‘Vetergesic’, Alstoe Veterinary, York, UK), diluted 1:10 in normal saline (0.9% NaCl) was then injected subcutaneously at an approximate dose of 0.1 mg/kg to provide postoperative analgesia. Tumors were monitored weekly using MRI by an independent observer. Animals were assigned randomly to treatment groups and animal survival was monitored by assessment of animal well-being every other day by monitoring weight (terminal weight loss considered at 20% of original body weight) and clinical signs of illness (ascites, reduced motion, ruffled fur, no response to external stimuli). One biological repeat was carried out.

### Syrian hamster intraperitoneally disseminated pancreatic cancer model

The Syrian hamster model of disseminated PaCa has previously been described.^28^ Syrian hamster studies were carried out at the Sino-British Research Center, Zhengzhou University.

SHPC6 cells (1×10^7^) were seeded into the lower right peritoneal cavity of Syrian hamsters. Four days later, 10 hamsters per group were injected intraperitoneally with 500 µL PBS or 2×10^7^ PFU virus on days 0, 2, 4. For hamster studies, VVL-21 armed with a human form of IL-21 was used. One biological repeat was carried out for each experiment. Animals were assigned randomly to treatment groups and animal survival was monitored by assessment of animal well-being every other day by monitoring weight (terminal weight loss considered at 20% of original body weight) and clinical signs of illness (ascites, reduced motion, ruffled fur, no response to external stimuli).

### Statistical analysis

All statistical analysis was undertaken in GraphPad Prism V.7. G*3 Power software was used to determine number of mice per group to see a 30% effect size. A power calculation based on an F analysis of variance (ANOVA) (setting parameters alpha=0.1; power=90%; effect size=30%, groups=3) was used. To compare different datasets, unpaired Student’s t-test, one-way or two-way ANOVA with Bonferroni post-test was used and results were expressed as mean±SEM. Survival data were represented in a Kaplan-Meier plot and log rank analysis was used to determine if differences between groups were significant. A value of p<0.05 (*) was considered as statistically significant.

## Results

### Modification of the VV genome can enhance systemic spread

**Figure 1:**
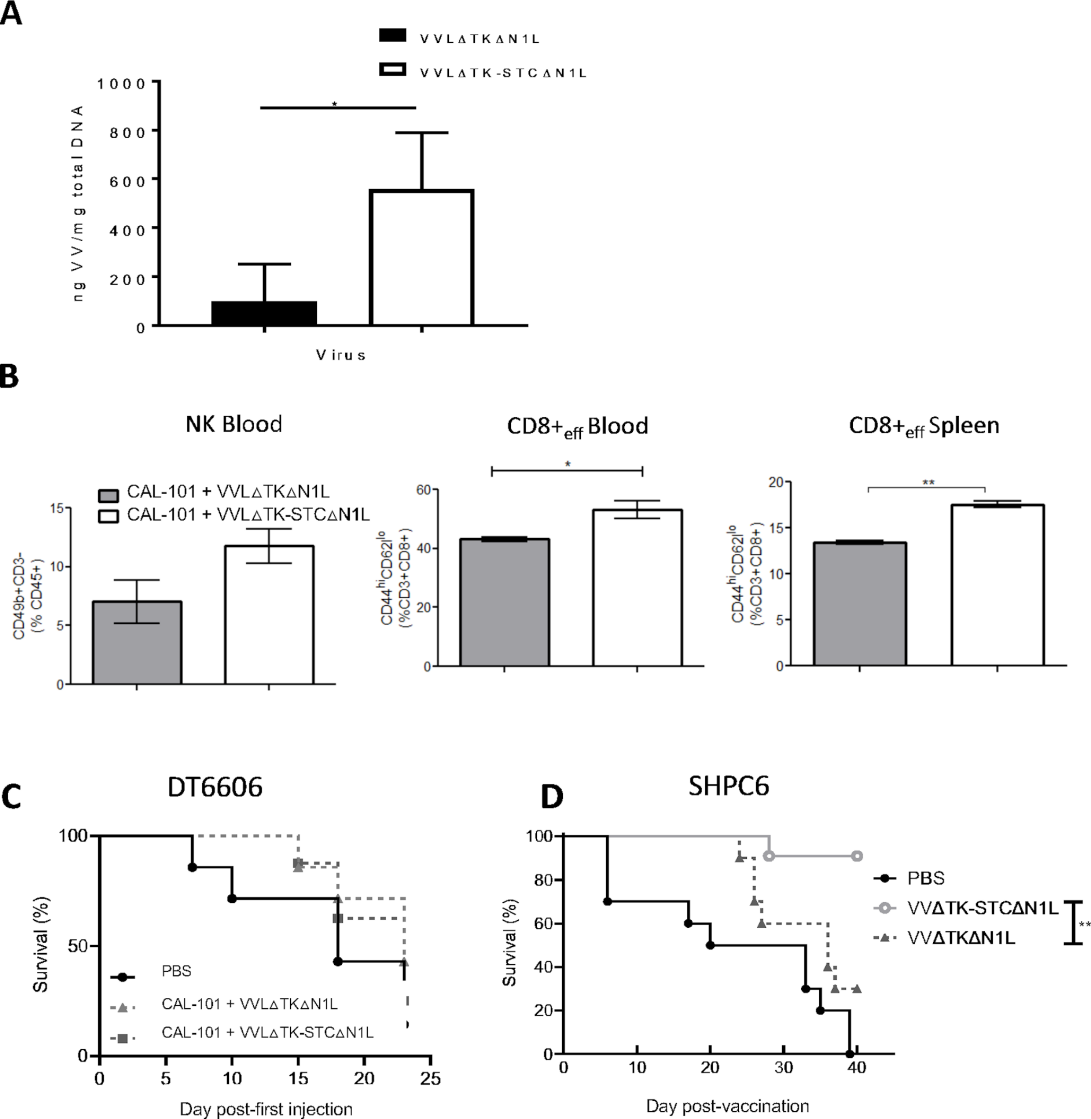
VVLΔTK-STCΔN1L is effective at reaching the tumor and inducing antitumor efficacy after CAL-101-potentiated systemic delivery. (A, B) DT6606 tumors were established subcutaneously in immunocompetent C57BL/6 mice. Once palpable (100 mm^3^), mice were treated with CAL-101 (10 mg/kg) by oral gavage followed 3 hours later by intravenous injection using 1×10^8^ plaque-forming unit (PFU)/injection VVLΔTKΔN1L that does not contain a modified second copy of B5R, or VVLΔTK-STCΔN1L. Treatments were given on days 0, 2 and 4. (A) Five days following the treatment, tumors were excised and viral load analyzed using quantitative PCR shown as ng viral DNA per mg total DNA (n=3/group). (B) Five days following the treatment, blood and spleen were analyzed using flow cytometry for natural killer (NK) or effector CD8+ T cell populations (n=3/group). Mean±SEM is shown and significance analyzed using an unpaired Student’s t-test. (C) DT6606 tumors were established and treated with CAL-101 (10 mg/kg) by oral gavage followed 3 hours later by intravenous injection using 1×10^8^ PFU/injection on days 0, 2 and 4 and 2×10^8^ PFU/injection on days 13, 15 and 17 and survival monitored. Kaplan-Meier survival analysis with log rank (Mantel-Cox) tests were used to assess survival (n=7–8/group). (D) SHPC6 tumors were established intraperitoneally in Syrian Hamsters. Hamsters were treated intraperitoneally with 2×10^7^ PFU/mL VVLΔTKΔN1L or VVLΔTK-STCΔN1L on days 4, 6 and 8 post-tumor implantation. Of note, no CAL-101 was delivered prior to intraperitoneal injection of virus. Kaplan-Meier survival analysis with log rank (Mantel-Cox) tests were used to assess survival (n=10/group). *P<0.05; **p<0.01. PBS, phosphate-buffered saline.

We have previousl demonstrated that a TK-deleted, N1L-deleted VV has potent efficacy and tumor selectivity in vivo^13 14^ (online supplemental figure S1). To maximize the oncolytic potential of VV, we enhanced the capacity for systemic spread by increasing EEV production. The VV B5R protein is critical for EEV formation, but deletion of the four short consensus repeat (SCR) domains within B5R has previously been shown to enhance viral EEV production.^29 30^ By removal of the SCR domains in B5R, we were able to enhance EEV production after virus replication in a TK-deleted Lister strain backbone (VVLΔTKΔB5RSCR) (online supplemental figure S2A), however improved comet tail formation evidencing EEV production came at the expense of attenuated total viral replication, reflected by the reduced plaque size (online supplemental figure S2B), as previously noted.^30^ We therefore retained the original B5R gene, but placed a second copy, in which the SCR domains were deleted, and only the signal peptide, stalk (S), transmembrane (T) and cytoplasmic tail (C) regions (STC) remained, under H5 promoter control within the TK region (VVLΔTK-STC) (online supplemental figure S2A). VVLΔTK-STC demonstrated improved EEV production after viral replication without loss of plaque size (online supplemental figure S2B). Our oncolytic VV platform (VVΔTKΔN1L)^13^ was then modified to overexpress B5R-STC in the TK region without disruption of the native B5R gene. In vitro, this virus demonstrated cytotoxicity in a panel of murine, hamster and human PaCa cell lines (online supplemental figure S2C) and improved replication and EEV release (online supplemental figure S2D–G). When delivered intravenously to established subcutaneous PaCa, using the PI3Kδ inhibitor CAL-101 to prevent macrophage uptake of the virus after intravenous injection as previously described,^16^ VVLΔTK-STCΔN1L (hereafter referred to as VV CTRL) was more effective than the parental virus at replicating and spreading within tumors (figure 1A) and resulted in a modest increase in circulating NK cells and circulating and splenic effector CD8+ T cells (figure 1B). Of note, expression of STC would not be expected to enhance initial viral delivery to tumors as laboratory viral manufacture results in IMV and not EEV production. Increased EEV production occurs only after viral replication within the tumor tissues. Efficacy of VV CTRL was assessed using an established subcutaneous DT6606 model. After CAL-101 administration and six intravenous injections, there was no discernible difference in survival (figure 1C). However, using a Syrian hamster model in which we can recapitulate disseminated PaCa (SHPC6), with neoplastic progression similar to end-stage human PaCa,^28^ animals treated intraperitoneally with VV CTRL survived significantly longer compared with VVLΔTKΔN1L-treated animals (figure 1D), suggesting that by enhancing the spread of VV we can improve the clinical prospects for VV-based OVT.

10.1136/jitc-2020-001624.supp2 Supplementary data

### Arming modified VV with IL-21 improves antitumor efficacy

To combat the immune suppressive TME and improve the in vivo efficacy associated with VV CTRL, the immunomodulatory cytokine IL-21 was incorporated into the N1L region of the virus under control of the H5 promoter as described previously.^25 27^ IL-21 expression, virus replication and cytotoxicity after VVL-21 infection was confirmed in murine, hamster and human PaCa cell lines that all supported virus replication and cytotoxicity (online supplemental figure S3A–F). In vivo, intratumoral accumulation of virus after intravenous delivery of VVL-21 to subcutaneous DT6606 pancreatic tumors was enhanced by pretreatment with CAL-101 administered by oral gavage 3 hours prior to viral delivery (online supplemental figure S4A, B). As noted previously,^16^ tumor growth was controlled more effectively after three intravenous injections potentiated by CAL-101 administration compared with administration without CAL-101 (online supplemental figure S4C). In vivo efficacy of VVL-21 (VVLΔTK-STCΔN1L-IL21) was compared with delivery of VV CTRL (VVLΔTK-STCΔN1L) to determine the effect of IL-21 on treatment efficacy.

**Figure 2:**
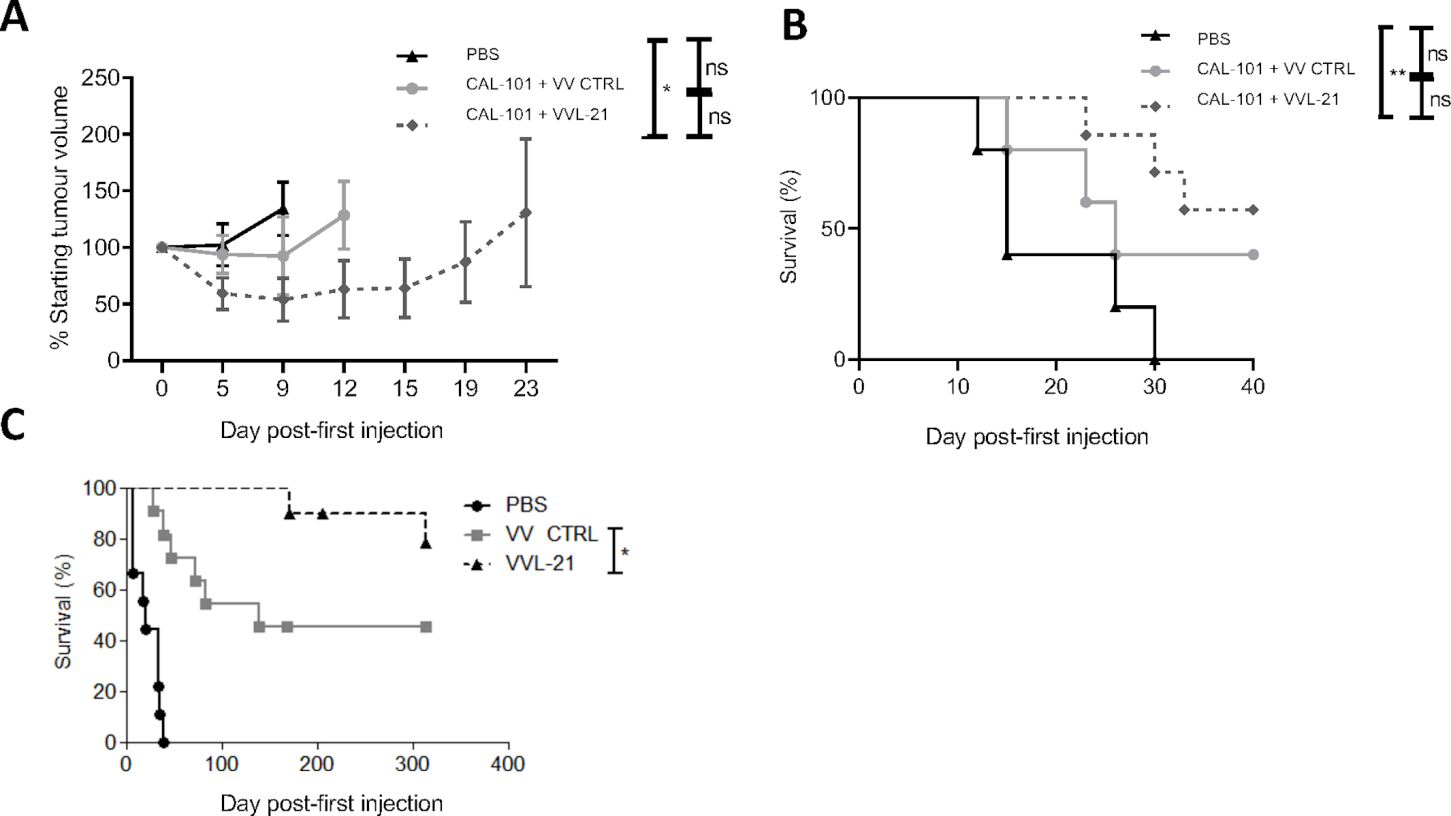
Arming VVLΔTK-STCΔN1L with interleukin (IL)-21 (VVL-21) improves in vivo antitumor efficacy in murine and hamster models of pancreatic cancer. (A, B) DT6606 tumors were established subcutaneously in immunocompetent C57BL/6 mice. Once palpable (100 mm^3^), mice were treated with CAL-101 (10 mg/kg) by oral gavage followed 3 hours later by intravenous injection using 1×10^8^ plaque-forming unit (PFU)/injection VVLΔTK-STCΔN1L-IL21 (VVL-21) or VV CTRL (no IL-21). Treatments were given on days 0, 2 and 4 (n=5–7/group). (A) Tumor size was monitored twice weekly and the mean±SEM is shown. Significance was assessed using a two-way analysis of variance with Tukey’s multiple comparison post-test and is shown for day 9. (B) Kaplan-Meier survival analysis with log rank (Mantel-Cox) tests were used to assess survival. (C) Disseminated SHPC6 tumors were established intraperitoneally in Syrian Hamsters. Hamsters were treated intraperitoneally with 2×10^7^ PFU/mL VV-CTRL or VVL-21 (in this case, the virus expressed a human version of IL-21) on days 4, 6 and 8 post-tumor implantation. Kaplan-Meier survival analysis with Gehen-Breslow-Wilcoxon tests were used to assess survival (n=9–11/group). *P<0.05; **p<0.01. ns, not significant; PBS, phosphate-buffered saline.

After three injections (1×10^8^ PFU on days 0, 2, 4), VVL-21 administration after CAL-101 delivery was able to slow tumor growth (figure 2A) and enhance overall survival (figure 2B) when compared with VV CTRL, although VVL-21 was unable to prevent tumor escape from control and by day 15, tumors in this group began to enlarge, suggesting further scope for improvement of the regime by increasing the number of doses administered and/or by combination with other immunomodulators. Intraperitoneal delivery of VVL-21 to the Syrian hamster model of disseminated PaCa also significantly enhanced survival compared with VV CTRL, producing a highly durable antitumor effect in this model (figure 2C).

### VVL-21 improves adaptive and innate immune responses in tumor-bearing animals

**Figure 3:**
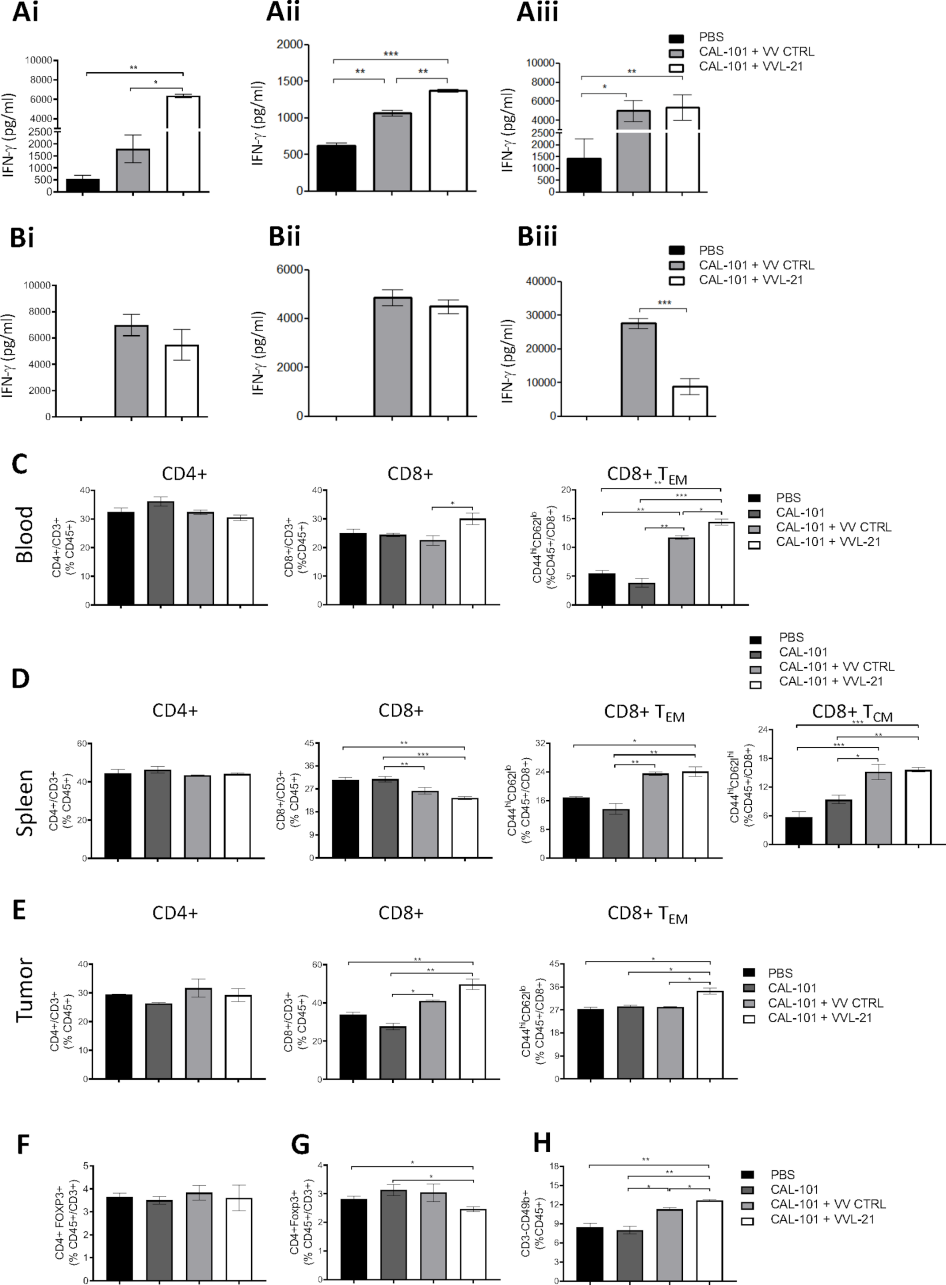
VVL-21 induces robust antitumor adaptive immune responses after systemic delivery. DT6606 tumors were established as previously and treated on days 0, 2 and 4 with 1×10^8^ plaque-forming unit (PFU) VV CTRL or VVL-21. (A–B) After treatment, the response of splenocytes to mitomycin C killed tumor cells (A) or viral protein (B8R epitope) (B) was examined ex vivo using tumor cell restimulation assays on days 7 (i), 10 (ii) or 12 (iii) after the first treatment. Interferon (IFN)-γ production in response to stimulation was determined after incubation for 72 hours using ELISA. Mean production±SEM is shown and a one-way analysis of variance (ANOVA) with Tukey’s multiple comparison post-test used to determine statistical significance (n=3/group). (C–H) 10 days after the first treatment, blood (C), spleen (D) and tumor (E) was collected and analyzed using flow cytometry for the presence of CD4+, CD8+, effector CD8+ (T_EM_) or central memory CD8+ (T_CM_) T cells. Mean populations±SEM are shown and a one-way ANOVA with Tukey’s multiple comparison post-test was used to determine statistical significance (n=3/group). (F–G) Intratumoral (F) and splenic (G) regulatory T populations were analyzed using flow cytometry 10 days after the first treatment. Mean populations±SEM are shown and a one-way ANOVA with Tukey’s multiple comparison post-test was used to determine statistical significance (n=3/group). (H) Natural killer (NK) cells responded rapidly to treatment and an elevation was detected in the blood using flow cytometry 4 days after the first treatment. Mean populations±SEM are shown and a one-way ANOVA with Tukey’s multiple comparison post-test was used to determine statistical significance (n=3/group). *P<0.05; **p<0.01; ***p<0.001. PBS, phosphate-buffered saline.

To determine the functional activity of VVL-21, established tumors were treated with CAL-101 3 hours prior to intravenous delivery of 1×10^8^ PFU VVL-21 or VV CTRL three times (days 0, 2, 4). Both viruses were able to significantly enhance ex vivo IFN-γ production by splenocytes stimulated with mitomycin C-treated DT6606 tumor cells harvested at 7, 10 and 12 days post-treatment. VVL-21-treated groups demonstrated a more rapid development of antitumor immunity (figure 3Ai–iii). Conversely, restimulation with the viral immunogen B8R demonstrated that both viruses induced antiviral immunity to the same extent at days 7 and 10, but antiviral immunity induced by VVL-21 reduced by day 12 post-treatment, suggesting an opportunity for re-administration of the virus after day 13 (figure 3B).

We next assessed adaptive immune cell populations in the tumor, spleen and blood 10 days following the first of three intravenous injections. Treatment with VV CTRL, VVL-21 or CAL-101 alone was unable to effect any changes in CD4+ T cell populations (figure 3C–E). However, circulating and intratumoral CD8+ T cell populations were significantly increased after viral treatment, in particular after treatment with VVL-21, but splenic CD8+ T cell populations were reduced, likely due to the mobilization to tumor sites. VVL-21 was also able to significantly expand the circulating, splenic and intratumoral effector CD8+ T cell subsets (CD8+ T_EM_) compared with VV CTRL (figure 3C–E). In addition, production of central memory CD8+ T cells (CD8+ T_CM_) was increased in response to viral treatment (figure 3D). As it has previously been reported that pharmacological inhibition of PI3 Kinase δ, as occurs following CAL-101 administration, can reduce TReg cell populations,^31^ we examined intratumoral TReg cells but we found that no treatment (CAL-101 alone or with viruses) impacted intratumoral TReg populations (CD4+FoxP3+) (figure 3F), however a treatment combination of CAL-101 with VVL-21 (but not VV CTRL) was able to reduce splenic TReg populations 5 days after treatment (figure 3G). In addition, NK cells responded rapidly to treatment and 4 days after the first injection, viral treatment enhanced circulating NK cells, with VVL-21 performing significantly better than VV CTRL (figure 3H). These results demonstrate that VVL-21 can manipulate both the adaptive (via CD8+ effector T cell induction) and innate (via NK cell induction) arms of the immune system and these modifications may provoke more robust antitumor immune effects.

### VVL-21 can re-educate macrophages to M1 phenotypes

**Figure 4:**
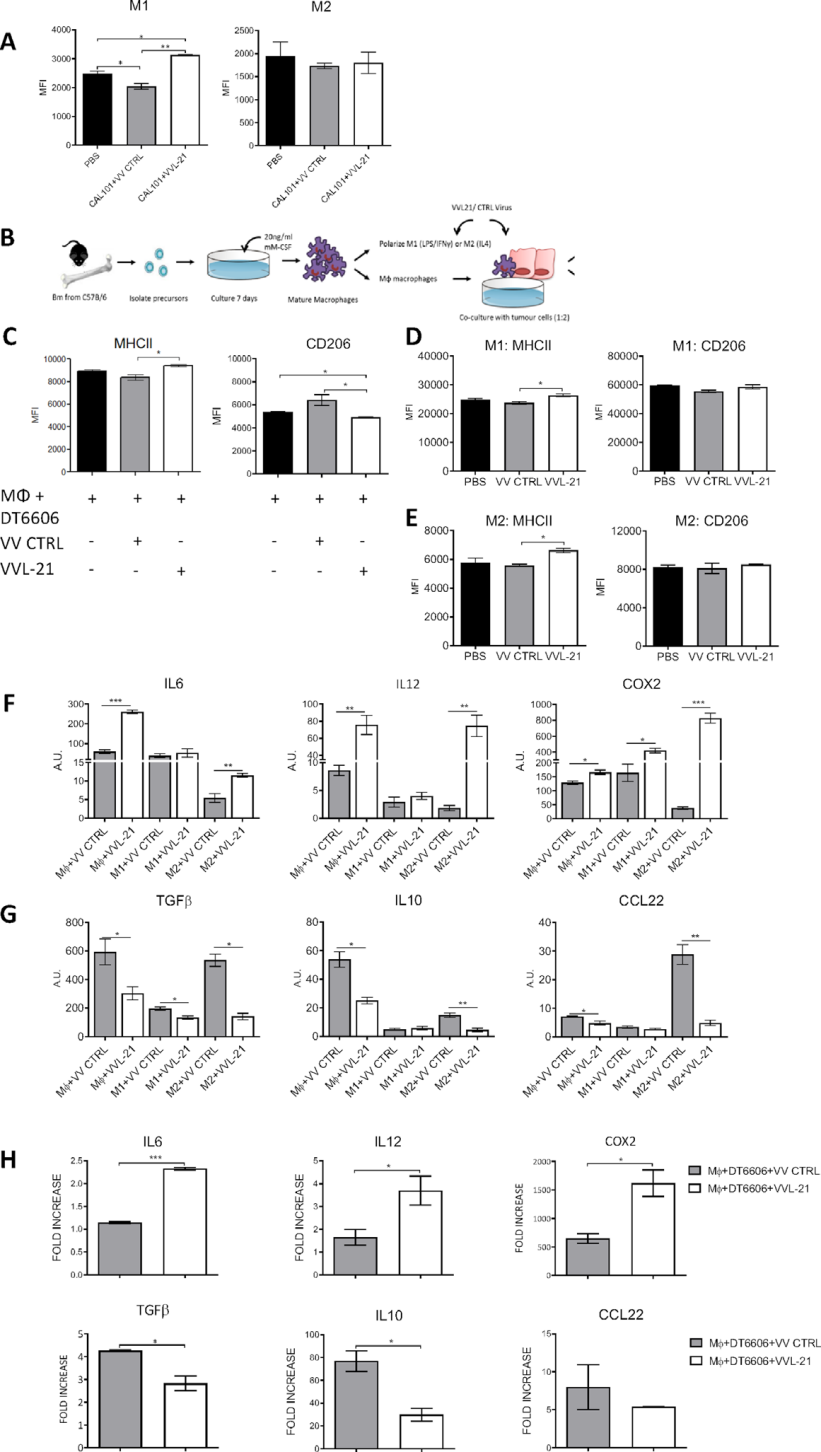
VVL-21 augments M1 macrophage polarization in vivo and in vitro. (A) DT6606 tumors were established as previously and treated on days 0, 2, 4 with 1×10^8^ plaque-forming unit (PFU) VV CTRL or VVL-21. Ten days after the first treatment, tumors were excised and macrophages analyzed using flow cytometry to determine major histocompatibility complex (MHC)II expression. MHCII^hi^ macrophages were considered M1 polarized and CD206^hi^ considered M2 polarized. Median fluorescence intensity (MFI)±SEM is shown and results analyzed using a one-way analysis of variance (ANOVA) with Bonferroni post-test (n=3/group). (B) Schematic detailing in vitro isolation and culture of bone marrow-derived macrophages. (C) Naïve macrophages were co-cultured with DT6606 tumor cells±VV CTRL or VVL-21. MHCII expression (for M1 phenotype) and CD206 expression (for M2 phenotype) were assessed using flow cytometry. MFI±SEM is shown and results analyzed using a one-way ANOVA with Bonferroni post-test (n=3/group). (D–E) Macrophages were polarized to an M1 () or M2 (E) phenotype and incubated with VV CTRL or VVL-21 at a multiplicity of infection (MOI) (of 1 PFU/cell for 24 hours. Expression of M1 (MHCII) and M2 (CD206) markers in each group was analyzed using flow cytometry. Mean MFI±SEM is shown and results analyzed using a one-way ANOVA with Bonferroni post-test (n=3/group). (F–G) Naïve, M1-polarized or M2-polarized macrophages were incubated with virus as above (MOI 1 PFU for 24 hours) and the expression of M1 markers (F) interleukin (IL)6, IL12 and COX2 or M2 markers (G) transforming growth factor (TGF)β, IL10 and CCL22 analyzed using quantitative PCR (qPCR). Arbitary Units (AU)±SEM is shown and results analyzed using a one-way ANOVA with Bonferroni post-test (n=3/group). (H) Naïve macrophages co-cultured with virus-infected DT6606 cells (using MOI of 1 PFU) were assessed for expression of M1 or M2 markers using qPCR 24 hours after incubation. The fold increase with respect to GAPDH is shown (±SEM) and results analyzed using a one-way ANOVA with Bonferroni post-test (n=3/group). *P<0.05; **p<0.01; ***p<0.001. PBS, phosphate-buffered saline.

Using the same model described above, innate immune compartments were examined at early (day 5, 1 day following the last treatment) and late (day 9, 5 days following the last treatment) time points (online supplemental figure S5). No significant differences in intratumoral polymorphonuclear leukocytes were seen at any time point (online supplemental figure S5A). Dendritic cell (DC) populations were increased in the tumor after treatment with both VV CTRL and VVL-21 at day 5, but populations had normalized to baseline (PBS) by day 9 (online supplemental figure S5A). There was no difference in macrophage M1 and M2 populations after both treatments compared with PBS-treated animals at early time points (online supplemental figure S5B), but an increase in M1 populations in mice treated with VVL-21 compared with both PBS group and VV CTRL group at day 9 was noted (figure 4A) using the gating strategies shown in online supplemental figure S5C–E. In vitro, VVL-21-infected DT6606 cells were able to increase expression of the M1 marker major histocompatibility complex (MHC)II and decrease expression of the M2 marker CD206 in co-cultured macrophages (figure 4B, C) and both M1-polarized (figure 4D) and M2-polarized (figure 4E) macrophages showed an increase in MHCII after infection with VVL-21 compared with VV CTRL. At the mRNA level, VVL-21 infection increased expression of M1 cytokine gene transcripts (*IL6*, *IL12* and *COX2*) (figure 4F) and reduced expression of M2 cytokine gene transcripts (*IL10*, *transforming growth factorβ* or *CCL22*) (figure 4G) in naïve, M1-polarized or M2-polarized macrophages or during naïve macrophages co-culture with infected DT6606 cells (figure 4H).

### VVL-21 sensitizes tumors to the checkpoint inhibitor α-PD1

**Figure 5:**
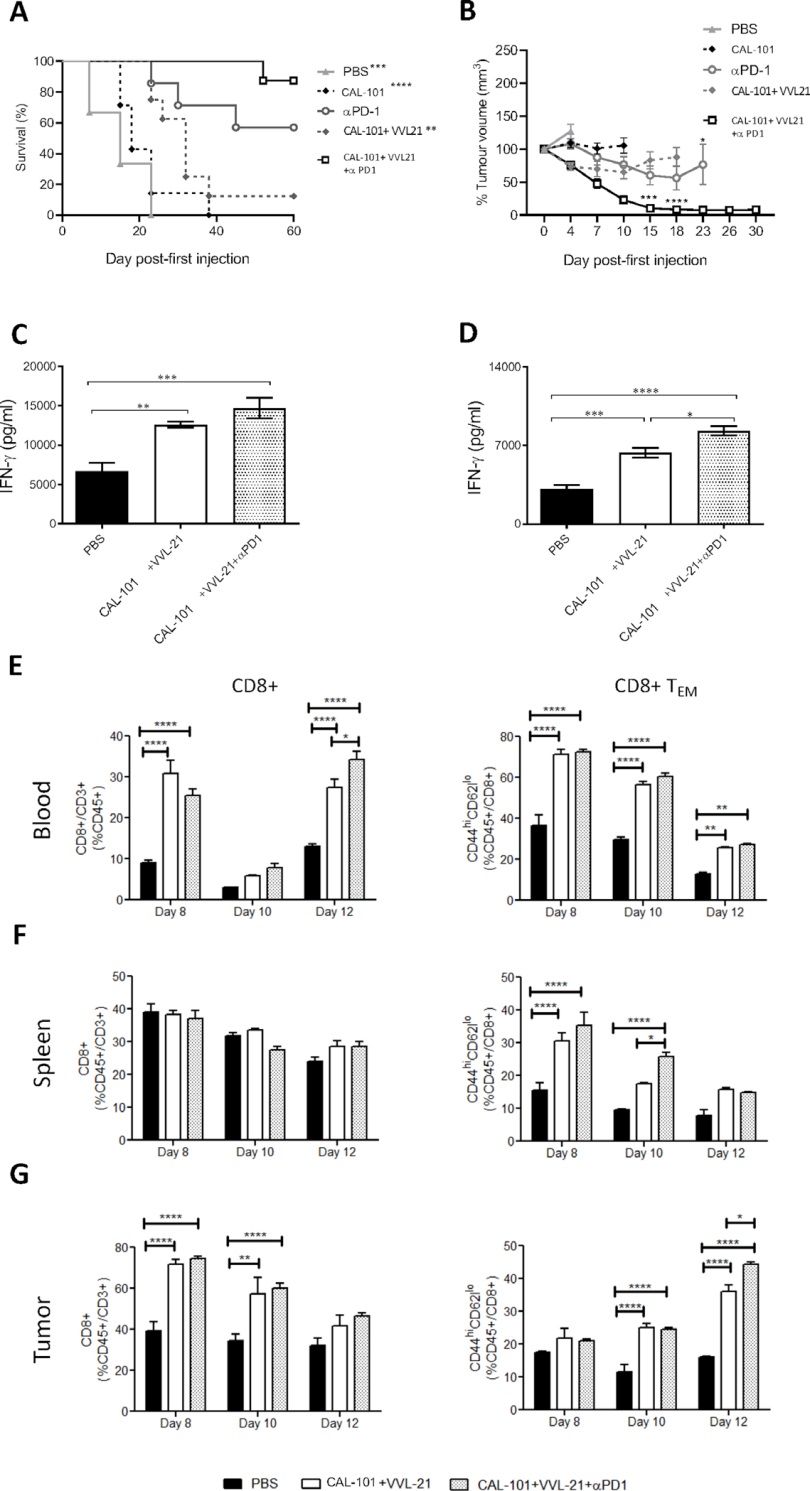
α-Programmed cell death protein 1 (α-PD1) can augment the antitumor efficacy of CAL-101 potentiated intravenous-delivered VVL-21 in vivo. (A–B) DT6606 subcutaneous tumors were established as previously and treated using 1×10^8^ plaque-forming unit (PFU) for injections on days 0, 2, 4 and 2×10^8^ on days 13, 15, 17. α-PD1 was administered by intraperitoneal injection on days 2, 5, 7, 15, 19 and 20 (200 µg/injection). (A) Kaplan-Meier survival analysis with log rank (Mantel-Cox) tests were used to assess survival. Significance in relation to CAL-101+VVL-21+α-PD1 group is shown (n=6–8/group). (B) Tumors were measured twice weekly and the mean tumor sixe (±SEM) plotted. A two-way analysis of variance (ANOVA) with Tukey’s multiple comparison post-test was used to measure significance. Significance of CAL-101+VVL-21+α-PD1 compared with CAL-101+VVL-21 is shown at days 15 and 18 and significance of CAL-101+VVL-21+α-PD1 compared with α-PD1 is shown at day 23. (C–G) DT6606 subcutaneous tumors were established as previously and treated using 1×10^8^ PFU for injections on days 0, 2, 4. α-PD1 was administered by intraperitoneal injection on days 2, 5 and 7 (200 µg/injection). (C, D) After treatment, the response of splenocytes to tumor cells was examined ex vivo using tumor cell restimulation assays on days 8 (C) or 10 (D) after the first treatment. Interferon (IFN)-γ production in response to stimulation was determined after 72 hours using ELISA. Mean production±SEM is shown and a one-way ANOVA with Tukey’s multiple comparison post-test used to determine statistical significance (n=3/group). (E–G) Eight, 10 and 12 days after the first treatment, blood (E), spleen (F) and tumor (G) was collected and analyzed using flow cytometry for the presence of CD8+ and effector CD8+ (T_EM_) cells. Mean populations±SEM are shown and a two-way ANOVA with Tukey’s multiple comparison post-test was used to determine statistical significance (n=3/group). *P<0.05; **p<0.01; ***p<0.001; ****p<0.0001.

Given the ability of VVL-21 to induce significant intratumoral effector CD8+ T cell responses, but its inability to exert long-term control over tumor growth, we investigated whether incorporation of a monoclonal antibody to PD-1 was able to enhance the antitumor efficacy of the regime. In vitro, we found that DT6606 cells expressed low levels of PD-L1, the natural ligand of PD1, however, levels were significantly increased after IFN-γ treatment (online supplemental figure S6A–D). Using an initial prime-only DT6606 in vivo study (injections on days 0, 2 and 4 following tumor growth to 100 mm^3^), we determined that the addition of α-PD1 administration improved the antitumor efficacy of the regime, but tumors began to re-grow from day 19 (online supplemental figure S6E). Thus, we investigated antitumor effect using the subcutaneous DT6606 tumor model, with a prime-boost regimen consisting of six viral injections (1×10^8^ PFU on days 0, 2, 4 and 2×10^8^ PFU on days 13, 15, 17) and six α-PD1 injections in an attempt to fully suppress tumor growth (online supplemental figure S7A). Of note, systemic delivery in the face of neutralizing antibody production after repeated injections has previously been shown to be possible.^8^ CAL-101-enhanced intravenous VVL-21 delivery was more effective compared with control groups at improving overall survival in this model (figure 5A), but combining viral treatment with α-PD1 treatment was able to significantly inhibit tumor growth compared with use of virus alone (figure 5B) and increased overall survival further (figure 5A). CAL-101 treatment alone was unable to control tumor growth. Similarly, treatment with α-PD1 alone was not able to control tumor growth, demonstrating the requirement for priming tumors to activate an immunogenic environment in order to sensitize them to this therapy.

Ex vivo splenocyte analysis demonstrated that at day 8 (4 days following the last of three treatments as shown in online supplemental figure S7B), CAL-101/VVL-21 enhanced splenocyte IFN-γ expression in response to mitomycin C-treated DT6606 tumor cells and inclusion of α-PD1 into the therapeutic regime improved IFN-γ induction (figure 5C), with the difference being more pronounced by day 10 (6 days following the last injection) (figure 5D). Functional analysis of CD8+ T cells demonstrated that treatment combinations involving VVL-21 were able to induce significant systemic CD8+ T cell response (figure 5E) and consistently enhanced effector CD8+ T cell populations in the blood, spleen and at later time points, the tumor (figure 5E–G). The added effect of α-PD1 on the generation of effector CD8+ T cells was obvious by day 10 post-treatment in the spleen (figure 5F) and by day 12 in the tumor (figure 5G), with significantly more effector T cells produced when this antibody was incorporated into the CAL-101/VVL-21 regime.

### Combining CAL-101, VVL-21 and α-PD1 creates the most effective systemically deliverable therapeutic regime

**Figure 6:**
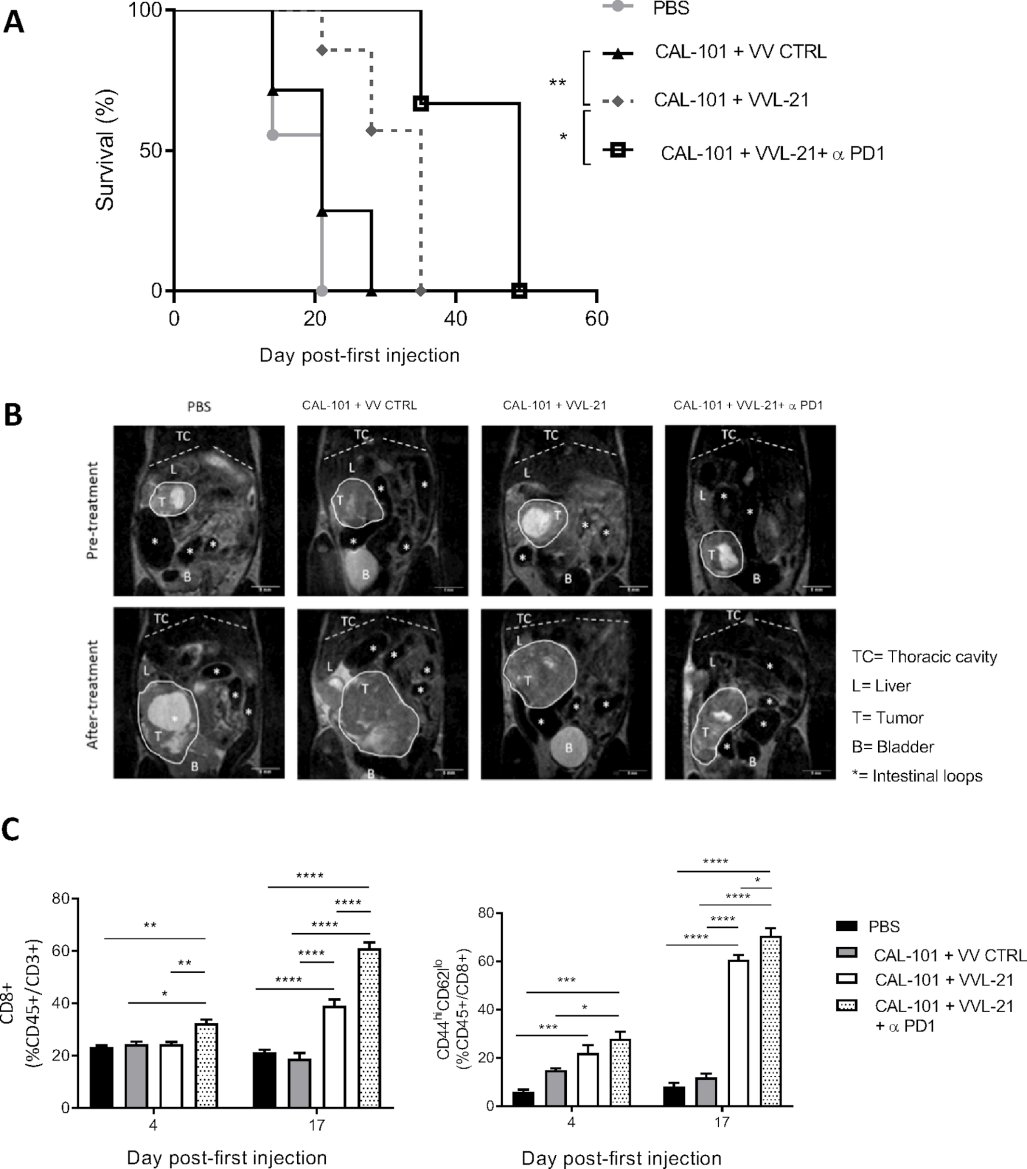
CAL-101-potentiated intravenous delivery of VVL-21 with concurrent α-programmed cell death protein 1 (α-PD1) treatment improves survival in a murine orthotopic model of cancer. Immunocompetent C57BL/6 mice had DT6606 tumor cells implanted in the tail of the pancreas. Ten days postimplantation, mice were treated according to the regime indicated in online supplemental figure S7, using 1×10^8^ plaque-forming unit (PFU)/injection on days 0, 2, 4 and 2×10^8^ PFU/injection on days 13, 15, 17. (A) Kaplan-Meier survival analysis with log rank (Mantel-Cox) tests were used to assess survival. Phosphate-buffered saline (PBS) n=9/group: VV CTRL n=7/group; VVL-21 n=7/group; VVL-21+α-PD1 n=3/group. *P=0.0355; **p=0.0084. (B) Representative images of MRI scanning that was conducted weekly. Pretreatment scans occurred 10 days post-tumor implantation. After treatment refers to scans taken in the last week of treatment. (C) Blood was drawn from the tail vein of mice at days 4 and 17 after the first day of treatment and analyzed for CD8CD8+ T cells and effector CD8+ T cells using flow cytometry. A two-way analysis of variance and Bonferroni post-test was used to determine statistical significance at each time-point. *P<0.05; **p<0.01; ***p<0.001; ****p<0.0001.

To more accurately reflect the organ-specific TME that may hinder effective therapeutic efficacies, treatment was explored using a DT6606-based murine orthotopic murine model of PaCa, treated six times as shown in online supplemental figure S7A. Animals treated with the standard regime of CAL-101/VVL-21 survived for longer than mice injected with CAL-101/VV CTRL, however, the most effective treatment regime consisted of CAL-101/VVL-21/α-PD1 in which mice could survive for up to 50 days post-treatment (figure 6A), showing a reduced rate of tumor growth (figure 6B). Analysis of circulating blood during the treatment (day 4 and day 17 after the first injection) demonstrated that combining VVL-21 with α-PD1 antibody increases the presence of CD8+ T cells in the blood. Effector CD8+ T cells were also significantly enhanced after VVL-21 treatment and addition of α-PD1 to this regime augmented the response even further and remained in effect at day 17, confirming that this regime is able to generate a strong and durable immune response (figure 6C).

## Discussion

On infection of host cells, VV replication occurs rapidly to produce two infectious forms of virion: the IMV, retained within the cell until lysis, and an enveloped form that either remains attached to the cell surface as cell enveloped virus (CEV) or is released from the cell surface as EEV, a critical form for local and distant cell to cell spread.^32 33^ Functional analysis has demonstrated that while the VV B5R protein is required for formation of EEV, mutant viruses lacking the SCR domains within the protein actually produce more EEV. However, improved EEV production comes at the expense of localized spread,^29 30^ suggesting these domains are vital for retention of the virus at the membrane as CEV to mediate local cell-cell spread. We therefore introduce a mutant form of the viral B5R protein into the TK region, while retaining an unmodified parental B5R (VV CTRL). This virus demonstrated enhanced EEV production without compromise of the ability to spread locally to form plaques in vitro. In vivo, we demonstrated that this virus, when administered intravenously, was recovered in larger quantities from tumors than the parental virus delivered using the same regime. Given that the addition of the STC domain had no impact on the ability of the virus to replicate in vitro, we speculate that via improved intratumoral EEV production, VV CTRL had an improved ability to spread and infect neighboring and distant tumor cells after initial replication cycles were complete within the tumor. In vivo, the addition of the STC domain was not sufficient to significantly control tumor growth after intravenous delivery to DT6606 subcutaneous models, however after intraperitoneal delivery to the SHPC6 disseminated model in Syrian hamsters, the VV CTRL was able to efficiently control tumor growth. This discrepancy is likely due to the nature of the tumors in each case, with the hamster model representing a late-stage disseminated cancer that requires from the virus a more robust spreading ability to infect and control tumor growth and further dissemination. While we were able to detect higher numbers of VV particles after VV CTRL intravenous delivery to a more constrained TME, which is arguably less reflective of natural scenarios, within the subcutaneous tumor, it is likely that there is less scope for a virus optimized for distant spread to control tumors in this environment and additional modifications are required to most effectively control tumor growth.

An effective approach to enhance the ability of OVT platforms to induce antitumor immune responses is to include immuno-stimulatory transgenes in the viral vectors. A number of pro-immune transgenes have been examined in this context, including granulocyte-macrophage colony-stimulating factor (GM-CSF),^34 35^ IL-10^15^ and IL-12,^36^ which demonstrate varying degrees of efficacy and safety. GM-CSF, incorporated in the clinically approved herpes simplex virus (HSV) vector Imlygic^34^ has demonstrated an ability to enhance HSV-mediated eradiation of melanoma and other malignancies, however its inclusion in therapies to treat PaCa may be counterproductive as there is emerging evidence of its role as a growth factor in this disease.^37^ IL-12 is considered one of the most potent activators of the immune system, but when delivered intravenously systemic accumulation of IL-12 can result in the rapid development of lethal inflammatory syndrome.^36^ In contrast, IL-21 has been found safe for clinical application^23 38^ and has a range of immune-stimulatory activities that suggest it as a powerful candidate for combination with OVT. Here, we demonstrate that IL-21 significantly improves the therapeutic efficacy associated with VV CTRL treatment in PaCa models via beneficial remodeling of immune elements of the TME, importantly macrophages, and engagement of adaptive immune responses. Macrophages are a heterogeneous population of phagocytic cells with distinct functional properties. Broadly, macrophage populations can be considered to have alternatively activated, tumor-promoting M2-like or classically activated, antitumor M1 phenotypes, although their considerable plasticity gives rise to a number of intermediate phenotypes and a transitional ability.^39^ M2-polarized macrophages predominate in the TME and foster tumor progression by inhibition of antitumor immune responses and promotion of angiogenesis, tumor cell proliferation and metastasis.^40^ The importance of M2 macrophages is exemplified by their correlation with poor prognosis in PaCa and other malignancies.^41^ We demonstrated that treatment with VVL-21 was effective at re-polarization of M2 macrophages to an M1 phenotype in vitro. Moreover, this virus encouraged M1 polarization of naïve macrophages. These results were reflected in vivo using PaCa tumor models in which M1-polarized macrophages were recovered in greater quantities from VVL-21-treated tumors. There have previously been varying reports on the effect of IL-21 on macrophages, with some reports supporting our data that IL-21 can promote an M1 phenotype after direct intratumoral delivery,^42^ while others suggest IL-21 favors M1 to M2 polarization.^43 44^ Our data demonstrate that intravenous-delivered IL-21 via OVT manifests an antitumor macrophage response. Interestingly, while we demonstrated an improved antitumor response, we noted a reduced antiviral response 12 days after administration of VVL-21 that was not seen after administration of VV CTRL. These data suggest that tumor and viral antigen presentation pathways differ, with the possibility that M1 macrophages, stimulated by IL-21 are presenting tumor antigen. Other antigen-presenting cells such as DCs, on which IL-21 has previously been shown to have suppressive effects regarding maturation and stimulatory capacity,^45^ may in this context be responsible for viral antigen presentation. Dissection of these important pathways will be critical for future rational development of the platform, but a reduced antiviral effect may be an important factor in the overall efficacy of treatment, by allowing prolonged OVT activity. We also noted a demonstrable effect of IL-21 on NK cell populations. NK cells play a critical role in the elimination of MHCI-deficient tumors that may otherwise evade immune surveillance. We previously demonstrated that modulation of the VV backbone by deletion of the N1L gene could enhance systemic NK cell responses and prevent postsurgical tumor recurrence in murine models of pancreatic, lung and breast cancer^13^ and IL-21 is able to increase these responses further. Interestingly, Seo *et al* recently reported a critical role for IL-21 in the reversal of NK cell exhaustion, common to MHCI-deficient tumors,^46^ thus further phenotypic examination of the NK populations induced by VVL-21 is warranted in future experiments, although based on our results, this can reasonably be expected to extend to our model.

In addition to enhancing innate antitumor immune responses, VVL-21 also enhanced adaptive T cell immunity, with virus treatment consistently elevating both systemic and intratumoral effector and memory T cell populations and IL-21 exacerbating the effects on effector CD8+ T cell populations further. Interestingly, while VVL-21 treatment resulted in production of antitumor immunity as evidenced by splenocyte restimulation ex vivo using killed tumor cells to supply tumor antigens, the effect on antiviral immunity was less pronounced and of shorter duration, suggesting that our classical three-injection regime could be boosted by administration of further injections after antiviral T cell responses waned. As such, a prime-boost regime was developed in which virus was administered on days 0, 2 and 4 then 13, 15 and 17 in an attempt to maximize antitumor efficacy and prevent tumor re-growth post-treatment. The remodeling of the immune elements of the TME by VVL-21 suggests that treatment may increase the sensitivity of PaCa to ICI therapy, to which PaCa is inherently insensitive. The combination of OVT and ICI therapy is currently being explored for a number of cancers^47^ and indeed we demonstrated a potent synergy when α-PD1 treatment was introduced subsequent to systemic OVT.

Of note, neither CAL-101 nor α-PD1 demonstrated any efficacy when used independently in our regime. CAL-101 was unable to significantly alter intratumoural TReg levels, thought to be a major mechanism by which CAL-101 exerts its therapeutic effect in clinical treatment of chronic lymphocytic leukemia (CLL).^48 49^ It should be noted that during this study, CAL-101 was delivered purely as a mechanism to enhance systemic virus delivery to tumors and as such, used in lower doses and with less frequency than the regime applied therapeutically for treatment of CLL. Interestingly, it has recently been reported that PI3Kδ inhibition antagonizes ICI activity,^49^ however here we show these two agents can both potentiate antitumor activity of OVT, resulting in effective treatment and induction of antitumor immunity against PaCa.

Crucially, accepting the limitations of subcutaneous tumor models to accurately reflect the native TME, we analyzed treatment efficacy in more complex disease models; a disseminated Syrian hamster intraperitoneal model in which tumor progression is similar to end-stage human PDAC^28^ and an orthotopic DT6606 murine model, in which the organ-specific TME is more accurately modeled compared with a subcutaneous model. We demonstrate potent antitumor efficacy in these complex disease models after systemic administration, which overcomes a significant limitation of OVT that restricts current use to intratumorally injectable lesions.

Together, these results describe a rationally constructed OV-based therapeutic platform that effectively addresses many of the shortfalls of current OV-based platforms in clinical development and may expand the therapeutic landscape for ICI treatments.

## Data Availability

Data are available in a public, open access repository. Data are available on reasonable request. In this manuscript, we have not created a data base, however, the materials such as viral vectors will be available on a reasonable request based on an appropriate MTA.
