# Correction: *A systemically deliverable Vaccinia virus with increased capacity for intertumoral and intratumoral spread effectively treats pancreatic cancer*

**DOI:** 10.1136/jitc-2020-001624corr1

**Published:** 2021-10-11

Marelli G, Chard Dunmall LS, Yuan M, *et al*. A systemically deliverable Vaccinia virus with increased capacity for intertumoral and intratumoral spread effectively treats pancreatic cancer. *J ImmunoThera Cancer* 2021;9:e001624. doi:10.1136/jitc-2020-001624

This article has been corrected since it first published. The provenance and peer review statement has been added.

