# Supplementary material for: A systemically deliverable Vaccinia virus with increased capacity for intertumoral and intratumoral spread effectively treats pancreatic cancer
Source: J Immunother Cancer. 2021 Jan 22;9(1):e001624. doi: 10.1136/jitc-2020-001624 (PMC7839893; doi:10.1136/jitc-2020-001624)
Supplement: Supplementary data [file jitc-2020-001624supp001.pdf]

A systemically deliverable Vaccinia virus with increased capacity for inter- and intra-tumoral spread effectively treats pancreatic cancer

Short title: Systemically delivered Vaccinia virus treats pancreatic cancer

Giulia Marelli[1]†, Louisa S. Chard Dunmall[1]†, Ming Yuan[1], Carmela Di Gioia[1], Jinxin Miao[2], Zhenguo Cheng[2], Zhongxian Zhang[2], Peng Liu[1], Jahangir Ahmed[1], Rathi Gangeswaran[1], Nicholas R. Lemoine[1, 2]* and Yaohe Wang[1, 2]*

Supplementary Materials and Methods

Cell lines and cell culture

Macrophages were cultured in Iscove's Modified Dulbecco's Medium (IMDM) with 10% foetal bovine serum (FBS). T cell culture medium (TCM) was RPMI-1640, 10% FBS, 1% streptomycin, 1% penicillin, and 1% sodium pyruvate. The murine pancreatic ductal adenocarcinoma (PDAC) cell line DT6606 was cultured from LSL-Kras$^{G12D/+}$; Pdx-1-Cre mice that had developed PDAC. The TB11381 PDAC cell line was cultured from LSL-Kras$^{G12D/+}$/TRP53$^{R172H/+;}$ Pdx-1-Cre mice that had developed PDAC [1]. These were kindly provided by David Tuveson (Cancer Research UK Cambridge Research Institute, now at Cold Spring Harbor Laboratory) and maintained in Dulbecco's Modified Eagles Medium (DMEM) supplemented with 10% FBS. CV1 (African monkey kidney) cells were obtained from American Type Culture Collection (ATCC, VA, USA) and grown in DMEM supplemented with 10% FBS. The Syrian hamster cell lines HAP-T1 and HPD-1NR (pancreatic carcinoma) were obtained from the German collection of microorganisms and cell cultures and maintained in DMEM and RPMI respectively, supplemented with 10% FBS. SHPC6 was maintained in Dulbecco’s modified Eagle’s medium (DMEM) with 10% FBS was kindly provided by W. Wold

(St. Louis University, St. Louis, MO, USA). The human pancreatic carcinoma cell lines SUIT2 and Mia-PaCa2 cell lines were obtained from Cancer Research UK central cell services (CRUK CCS, Clare Hall, Herts, UK) and maintained in DMEM supplemented with 10% FBS. CT26 (metastatic colon adenocarcinoma) and LLC (Lewis lung carcinoma) were obtained from the Cancer Research UK central cell bank (CRUK, Clare Hall, Herts, UK) and maintained in DMEM supplemented with 10% FBS Cell lines were routinely tested for mycoplasma and were maintained at 37°C with 5% CO_2.

Reagents

The selective PI3Kδ inhibitor CAL101 was purchased from Selleckchem, re-suspended at 30mg/ml with 30% PEG 400, 0.5% Tween 80, 5% Propylene glycol and administered via oral gavage at 10mg/kg. The VV antibody used for the immuno-histochemical (IHC) experiments was a rabbit anti-VV polyclonal antibody supplied by AbD Serotec, batch number 250906, and was diluted 1:400. α-PD1 antibody (RMP1-14) was purchased from Bioxcell and injected i.p at a final dose of 200μg/mouse. Murine recombinant IFN-γ was purchased from Peprotech and used at the concentrations indicated in the results. FACS buffer was phosphate-buffered saline Dulbecco + 2 mM EDTA + 1% FBS. FACS antibodies were purchased from eBioscence (CD4 Alexa Fluor 700, CD62L PE-Cyanine7, CD45 eFluor 450, GR1 Afluor700, CD80 eFluor 450, CD3e PerCP- Cyanine5.5 B220 FITC, CD49b APC, CD11c PE), BD (CD8a PE), and Biolegend (CD44 Brilliant Violet, CD206 (MMR) PerCP/Cy5.5 CD11b FITC, F4/80 Brilliant Violet 650, MHCII PE –dazzle, FOXP3 Alexa Fluor 488, PD-L1 PE, Zombie NIR™, Zombie AQUA™). FOXP3 staining was carried out using True-Nuclear™ Transcription Factor Buffer Set from Biolegend. Recombinant mouse macrophage colony-stimulating factor (M-CSF), IL-4, IL-13 and IFN-γ were supplied by R&D Systems. LPS was supplied from Sigma-Aldrich.

Vaccinia virus replication assay

Appropriate cell lines were seeded in triplicate and infected 16 hours later with virus at a multiplicity of infection (MOI) of 1 PFU/cell. Cells and supernatant (or supernatant only to assess EEV release) were collected at 24, 48 and 72 hours post-infection and titers were determined by measuring the median tissue culture infective dose (TCID50) on indicator CV1 cells. Cytopathic effect was determined by light microscopy 10 days after infection. The Reed–Muench mathematical method was used to calculate the TCID50 value for each sample [2]. Viral burst titers were converted to PFU per cell based on the number of cells present at viral infection. One-way ANOVA followed by Bonferroni post-test was used to assess significance.

Cell cytotoxicity assay

The cytotoxicity of the viruses in each cell line was assessed in triplicate 6 days after infection with virus using an MTS non-radioactive cell proliferation assay kit (Promega) according to the manufacturers' instructions. Cell viability was determined by measuring absorbance at 490nm using a 96-well plate absorbance reader (Dynex) and a dose response curve created by non-linear regression allowing determination of an EC50 value (dose required to kill 50% of cells) as previously described [3].

Detection of IL-21 expression by ELISA

Appropriate cell lines were seeded in triplicate and infected 16 hours later with virus at a multiplicity of infection (MOI) of 1 PFU/cell. Supernatant was collected at 18, 24 and 48 hours post-infection and IL-21 levels measured using ELISA; Mouse IL-21 ELISA Ready-SET-Go! Kit; eBioscience or Human IL-21 ELISA Ready-SET-Go! Kit, 2nd Generation; eBioscience.

Expression of PD-L1 by FACS

$1x10^5$ DT6606 cells were added to FACS tubes in 500µl DMEM containing the appropriate units/ml (U/ml) IFN-γ. After 48 hours, cells were centrifuged and re-suspended in 30%

trypsin/10mM EDTA/PBS. Cells were incubated for 10 minutes at 37°C and then vortexed, centrifuged and the supernatant removed. Cells were then re-suspended in FACS buffer and processed for FACS analysis as described. Conditions were evaluated in triplicates.

Quantitative Polymerase Chain Reaction (qPCR)

Viral DNA extraction was performed using the Qiagen DNeasy Blood & Tissue Kit. Quantification of viral genome copy number was achieved using the TaqMan® PCR system provided by Applied Biosystems. For VV quantification, the primers and probe were designed for the Vaccinia virus late transcription factor 1 (VLTF-1) gene: Forward; 5'-AACCATAGAAGCCAACGAATCC, Reverse; 5'-TGAGACATACAAGGGTGGTGAAGT, Probe; ATTTTAGAACAGAAATACCC. The primers were supplied by Sigma-Aldrich. The standard was WT VV DNA, and 40ng of DNA was used per sample as the template. Viral genome copy number was normalized by total DNA loaded. Conditions were analysed in triplicate and two biological replicates were carried out.

Total RNA was extracted from cells using the RNeasy Mini Kit from Qiagen. Total RNA was re-suspended in nuclease-free water and quantified using the spectrophotometer NANODROP ND-1000. 1μg of RNA was reverse-transcribed using the High Capacity cDNA Reverse Transcription Kit (Applied Biosystems) following the manufacturer’s instructions. cDNA was analyzed through quantitative Real-Time PCR using the Fast SYBR Green system. Triplicate samples were analysed. Primers used were:

TGFβ_F 5’ TGGAGCAACATGTGGAACTC; TGFβ_R 5’ CGTCAAAAGACAGCCACTCA

IL12_F 5’ CTCCTGTGGGAGAAGCAGAC; IL12_R 5’ CAGATAGCCCATCACCCTGT

IL-10 F 5’ TGAATTCCCTGGGTGAGAAG; IL-10 R 5’ GCTCCACTGCCTTGCTCTTA

COX-2 F 5’ CCCCCACAGTCAAAGACACT; COX-2 R 5’ GGCACCAGACCAAAGACTTC

GAPDH F 5’ GGCATTGCTCTCAATGACAA; GAPDH R 5’ ATGTAGGCCATGAGGTCCAC

CCL22 F 5’ GCTCTCGTCCTTCTTGCTGT; CCL22 R 5’ GGGTGACGGATGTAGTCCTG

IL-6 F 5’ CCGGAGAGGAGACTTCACAG; IL-6 R 5’ AGAATTGCCATTGCACAAC

Splenocyte preparation

Harvested spleens were flushed through 70µm BD Falcon™ cell strainers with complete T-cell media (RPMI medium 1640 (Sigma Aldrich), 10% FCS, 1% streptomycin/ penicillin, 1% sodium pyruvate and 1% non-essential amino acids (Gibco®) and 0.1% β-mercaptoethanol). Red blood cells were lysed using RBC lysis buffer (Sigma-Aldrich) and re-suspended in complete T-cell medium.

Tumor cell preparation

Tumor cell suspensions were prepared by incubation with 2mg/ml collagenase plus 0.1mg/ml hyaluronidase for 30 minutes at 37°C. Cells were separated using a 70-mm cell strainer and re-suspended in complete T-cell medium.

Immunophenotyping of splenocytes and tumors

Single cell suspensions of tumor, spleen or blood were prepared in FACS buffer (FB) as described above. All fluorophore-conjugated antibodies used at 1:200 dilutions. Cells were blocked with anti-CD16/32 prior to incubation with fluorophore-conjugated antibody for 30 minutes. Cells were fixed in 2% formalin and analyzed using an LSRFortessa™ multichannel flow cytometer (Beckton Dickinson (BD) Biosciences). Raw data were analyzed using FloJo v10 (FloJo, LLC). Three samples/group were analysed and three biological repeats carried out.

Imaging

Magnetic Resonance Imaging (MRI) was performed once a week starting 10 days after tumor injection. Mice were anesthetized as described above and T2 image acquired using ParaVision software. Images were analyzed using VivoQuant software by an independent researcher.

Generation of Bone Marrow Derived Macrophages (BMDMs)

Bone marrow was harvested from femur of 8- to 12-week old mice by flushing the marrow out with IMDM supplemented with 10% Fetal Calf Serum (FCS) and ACK (100uL/bone) to lyse red blood cells. Flushed cells were re-suspended in 20mL IMDM, 10% FCS, 1% Penicillin/Streptavidin, 1% Glutamine and left to adhere overnight at 37°C, 5% CO2. After incubation, cell supernatant was removed, non-adherent cells were spun down. Cells were re-suspended at the concentration of $0.5x10^6$ cells/mL in complete bone marrow macrophage medium (IMDM, 10% FCS, 20 ng/mL M-CSF) using a suspension culture dish (Corning) (10mL/dish). After 7 days, cells were lifted and re-plated for stimulations and analysis. Cells were plated at the final concentration of $2.5x10^5$ cells/mL in 24 wells plate and stimulated with LPS (100ng/ml) and IFN-γ (20ng/ml) or recombinant IL4 (20ng/ml) for 24h. Cells were then washed with PBS and infected with virus using an MOI of 1PFU/cell for 24h. For tumor cell addition, DT6606 cells, at a ratio of 1 macrophage: 2 tumor cells were added to macrophages 4h before the virus infection. After 24h, cells were collected and lysed to recover RNA or used for FACS analyses. Samples were seeded in triplicate and two biological repeats carried out.

Ex-vivo **splenocyte re-stimulation**

Splenocytes were prepared as described and re-suspended in TCM to a final concentration of $5x10^6$ cells/ml. DT6606 were growth arrested using Mitomycin C (Roche) at a final concentration of 100mg/ml in a humidified incubator at 37°C with 5% CO_2 for 2 h. Cells were washed twice with PBS and re-suspended in TCM at a final concentration of $5x10^5$ cells/ml. 100µl aliquots of splenocyte suspensions were co-cultured with 100µl of Mitomycin C-treated cells in a 96-well plate. Separately, in order to assess immune response to the viruses, 100 µl aliquots of splenocyte suspensions were treated with Vaccinia virus B8R peptide (H-

2Kb/SIINFEKL, supplied by ProImmune), at a concentration of 5ng/ml in a 96-well plate. Plates were incubated at 37°C with 5% CO_2 for 3 days, centrifuged at 1200 rpm for 5 min and the supernatant collected. The concentration of IFN-γ was determined using a murine-specific IFN-γ ELISA kit from Biolegend. Samples were analysed in triplicate and three biological repeats carried out.

Immunohistochemistry

Appropriately harvested samples were immediately fixed in 4% w/v formalin (diluted with PBS) for 48h and transferred to a 70% ethanol solution. Samples were embedded in paraffin. Blocking, embedding, sectioning, antigen retrieval and immunohistochemistry staining were performed by Pathology Services, Barts Cancer Institute.

Assessment of virus biological distribution following i.v delivery into tumor bearing mice

Following the establishment of subcutaneous CT26 or LLC syngeneic flank tumours, $1x10^8$ PFU (in 50µl PBS) of either VVLΔTK or VVLΔTKΔN1L was injected via tail vein. One day later, three mice from each group were sacrificed via CO_2 inhalation. Tumor, brain, lung, liver, spleen, kidneys, heart, blood and ovaries were harvested. These were immediately snap-frozen in precooled (to -80°C) isopentane. Samples were subsequently thawed, weighed and homogenised using an Ultra-Turrax® homogeniser (at 12000 rpm) in a small volume of serum-free DMEM. Samples were diluted five-fold w/v (i.e. 5 µl DMEM per mg). After a further freeze-thaw cycle, tissue homogenates were titrated for live viral PFUs using the TCID50 assay.

Supplementary Methods References

1. Hingorani SR, Petricoin EF, Maitra A, et al. Preinvasive and invasive ductal pancreatic cancer and its early detection in the mouse. Cancer Cell. 2003;4(6):437-50.
2. Reed LJ, Muench H. A simple method of estimating fifty percent endpoints. The American Journal of Hygiene. 1938;27:493-497.
3. Wang Y, Hallden G, Hill R, et al. E3 gene manipulations affect oncolytic adenovirus activity in immunocompetent tumor models. Nat Biotechnol. 2003;21(11):1328-35.
