# Supplementary material for: A systemically deliverable Vaccinia virus with increased capacity for intertumoral and intratumoral spread effectively treats pancreatic cancer
Source: J Immunother Cancer. 2021 Jan 22;9(1):e001624. doi: 10.1136/jitc-2020-001624 (PMC7839893; doi:10.1136/jitc-2020-001624)
Supplement: Supplementary data [file jitc-2020-001624supp002.pdf]

A systemically deliverable Vaccinia virus with increased capacity for inter- and intra-tumoral spread effectively treats pancreatic cancer

Giulia Marelli[1†], Louisa S. Chard Dunmall[1†], Ming Yuan[1], Carmela Di Gioia[1], Jinxin Miao[2], Zhenguo Cheng[2], Zhongxian Zhang[2], Peng Liu[1], Jahangir Ahmed[1], Rathi Gangeswaran[1], Nicholas R. Lemoine[1, 2*] and Yaohe Wang[1, 2*]

Supplementary Figures

Supplementary figure 1

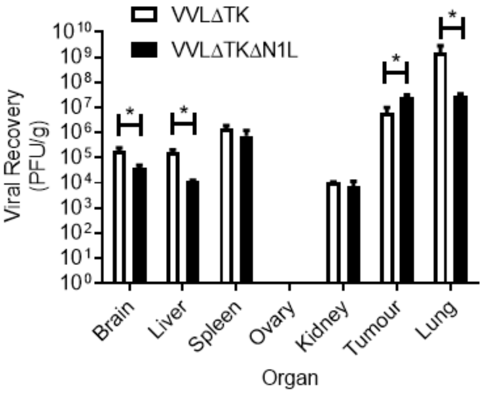

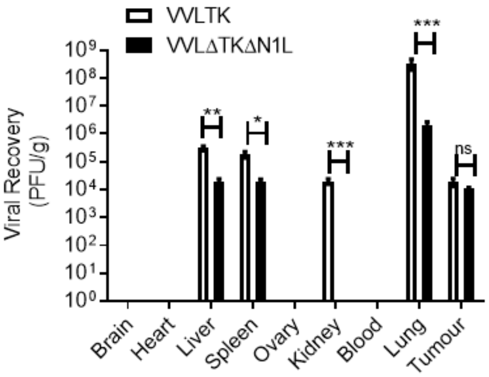

Supplementary figure 1. Deletion of the N1L gene of VVLΔTK improves the tumor selectivity of VV. Mice bearing syngeneic LLC (**A**) or CT26 (**B**) flank tumours (in C57BL/6 or BALB/c mice respectively) as indicated were administered tail vein injections of $1x10^8$ PFU of either VVLΔTK or VVLΔTKΔN1L. One day post injection, mice (n=3/group) were euthanized and organs were harvested. Viral titres in PFU/ gram weight of organ tissue (Y axes) were estimated using TCID50 assays on tissue homogenates. Unpaired T tests were used to determine statistical significance between each group in each organ.

Marelli G, *et al. J Immunother Cancer* 2021; 9:e001624. doi: 10.1136/jitc-2020-001624

Supplementary figure 2

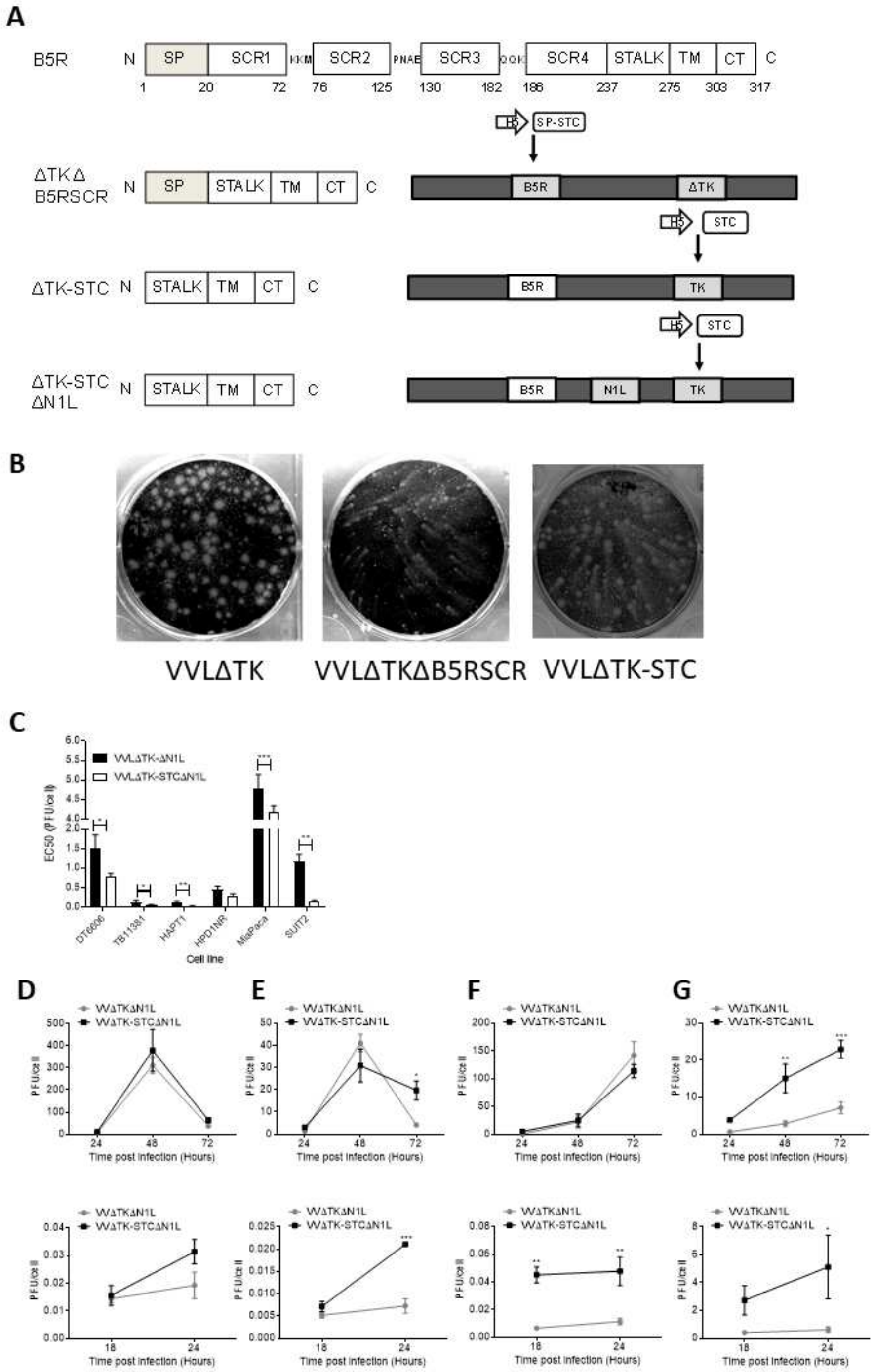

Supplementary figure 2. Insertion of the B5R STC domain into the TK region of VV can improve EEV production. Vaccinia virus Lister strain with deletions of the thymidine kinase (TK) and N1L genes, expressing IL-21 has been described previously as referenced in materials and methods. This virus was further modified by insertion of the B5R signal peptide, stalk (S), transmembrane (T) and cytoplasmic tail (C) into the T region as shown. (**A**) Schematic detailing the construction of mutant viruses. Light grey shaded areas indicate gene regions that were deleted in the final virus. The B5R STC domain was inserted into the viral TK region under control of the endogenous H5 promoter to drive high level, stable gene expression. (**B**) CV1 cells were seeded into 6 well plates and infected using 0.1 PFU/cell virus as indicated in the figure. 3 days post infection, cells were stained using crystal violet and photo scanned to reveal plaques and comet tails. (**C**) The EC50 value (dose required to kill 50% of cells) of VVLΔTK-ΔN1L and VVLΔTK-STCΔN1L was compared using MTS assays in murine (DT6606 and TB11381), hamster (HAPT1 and HPD1NR) and human (SUIT2 and MiaPaCa2) PaCa cell lines. Mean EC50 +/- SEM is shown and a Students unpaired T test used to assess significance (n=3/group). (**D-G**) Virus replication was determined in DT6606 (**D**), TB11381 (**E**), SUIT2 (**F**) and HPD1NR (**G**) cells over 72 hours. Virus production from whole cell lysates was determined (top graphs) and EEV production was determined via titration of the viral supernatant from the same experiments (bottom graphs). Mean PFU/cell +/- SEM is shown at each time-point and statistical significance determined using a one-way ANOVA and Tukey's multiple comparison post-test (n=3/group). *p<0.05; ** p<0.01; ***p<0.001.

Supplementary figure 3

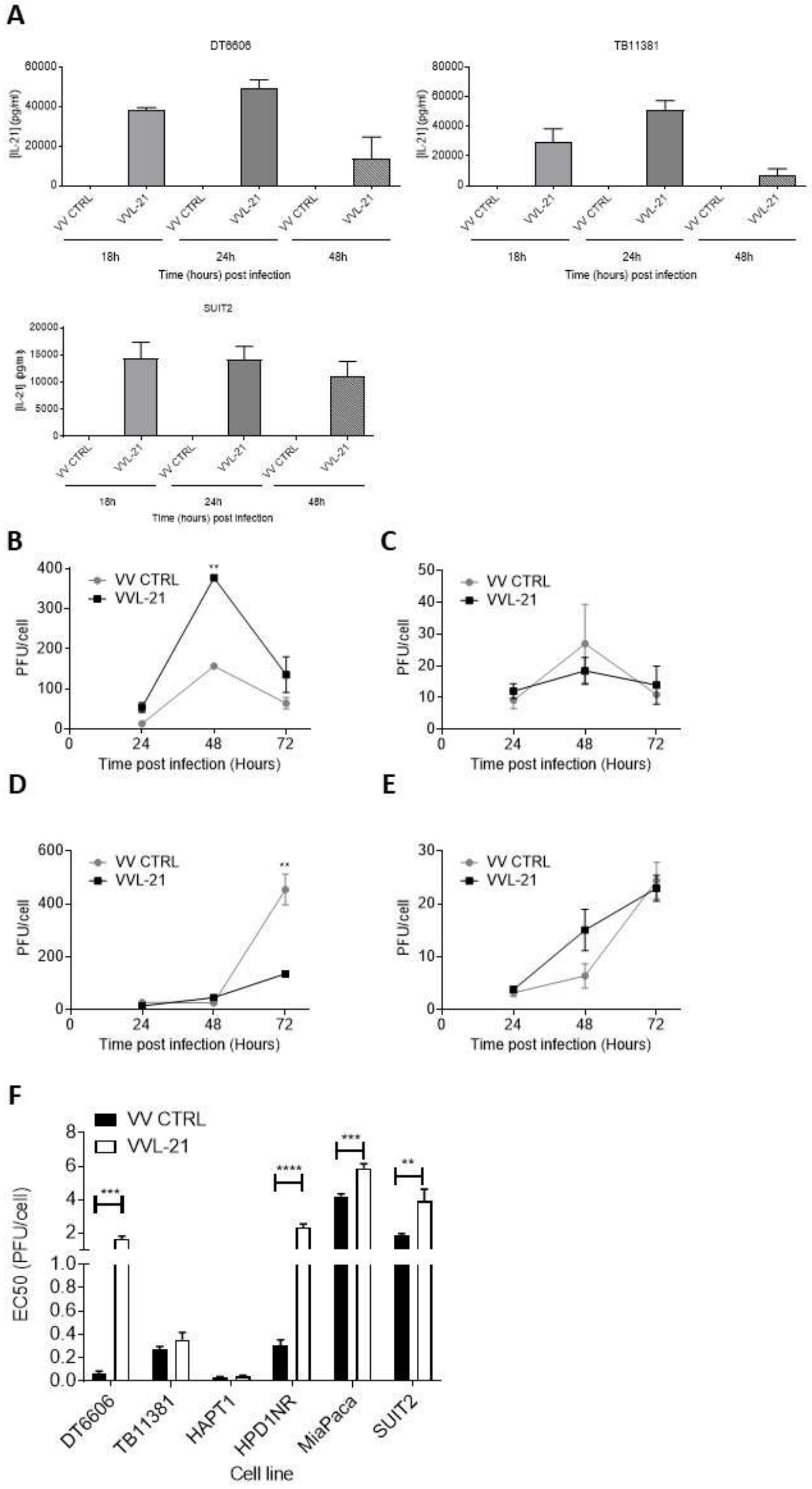

Marelli G, *et al. J Immunother Cancer* 2021; 9:e001624. doi: 10.1136/jitc-2020-001624

Supplementary Figure 3. VVL-21 remains cytotoxic and replication competent in murine, hamster and human PaCa cell lines. (**A**) Murine IL-21 expression was detected in supernatant from the indicated cell lines at 18, 24 and 48 hours post infection of 1PFU/cell VV CTRL or VVL-21 virus using ELISA (n=3/group). (**B-E**) Virus replication was determined in DT6606 (**B**), TB11381 (**C**), SUIT2 (**D**) and HPD1NR (**E**) cells over 72 hours. Mean PFU/cell +/- SEM is shown at each time-point and statistical significance determined using a one-way ANOVA and Tukey's multiple comparison post-test (n=3/group). (**F**) The EC50 value (dose required to kill 50% of cells) of VV CTRL and VVL-21 was compared using MTS assays in murine (DT6606 and TB11381), hamster (HAPT1 and HPD1NR) and human (SUIT2 and MiaPaCa2) cell lines. Mean EC50 +/- SEM is shown and a Students unpaired T test used to assess significance (n=3/group). *p<0.05; ** p<0.01; ***p<.0.001; ****p<0.0001.

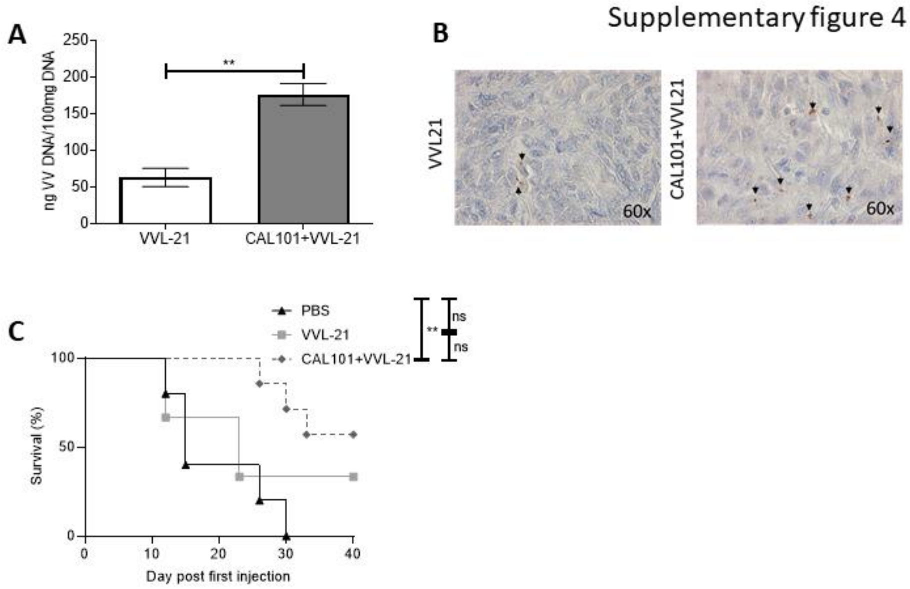

Supplementary Figure 4. Pre-treatment with CAL-101 improves the accumulation of VV in tumors after i.v delivery. (**A-B**) DT6606 tumors were established subcutaneously and once palpable (100mm^3) treated with CAL-101 or vehicle buffer, 3 hours prior to i.v injection of VVLΔTK-STCΔN1L-IL21 (VVL-21) (1x10^8 PFU/injection) on days 0, 2 and 4 (n=3/group). 5 days later, tumors were excised and viral load analyzed using qPCR (ng viral DNA per 100mg total DNA is shown) (**A**) or immuno-histochemical staining of tumor sections using a polyclonal α-VV antibody. (**B**). Mean +/- SEM is shown and significance analyzed using a Students unpaired T test. (**C**) DT6606 tumors were established subcutaneously and once palpable (100mm^3) treated with CAL-101 or vehicle buffer, 3 hours prior to i.v injection of VVLΔTK-STCΔN1L-IL21 (VVL-21) (1x10^8 PFU/injection) on days 0, 2 and 4 (n=3-5/group). Tumor size was monitored twice weekly and the mean +/- SEM is shown. Significance was assessed using a two-way ANOVA with Tukey's multiple comparison post-test. *p<0.05; ** p<0.01.

Supplementary figure 5

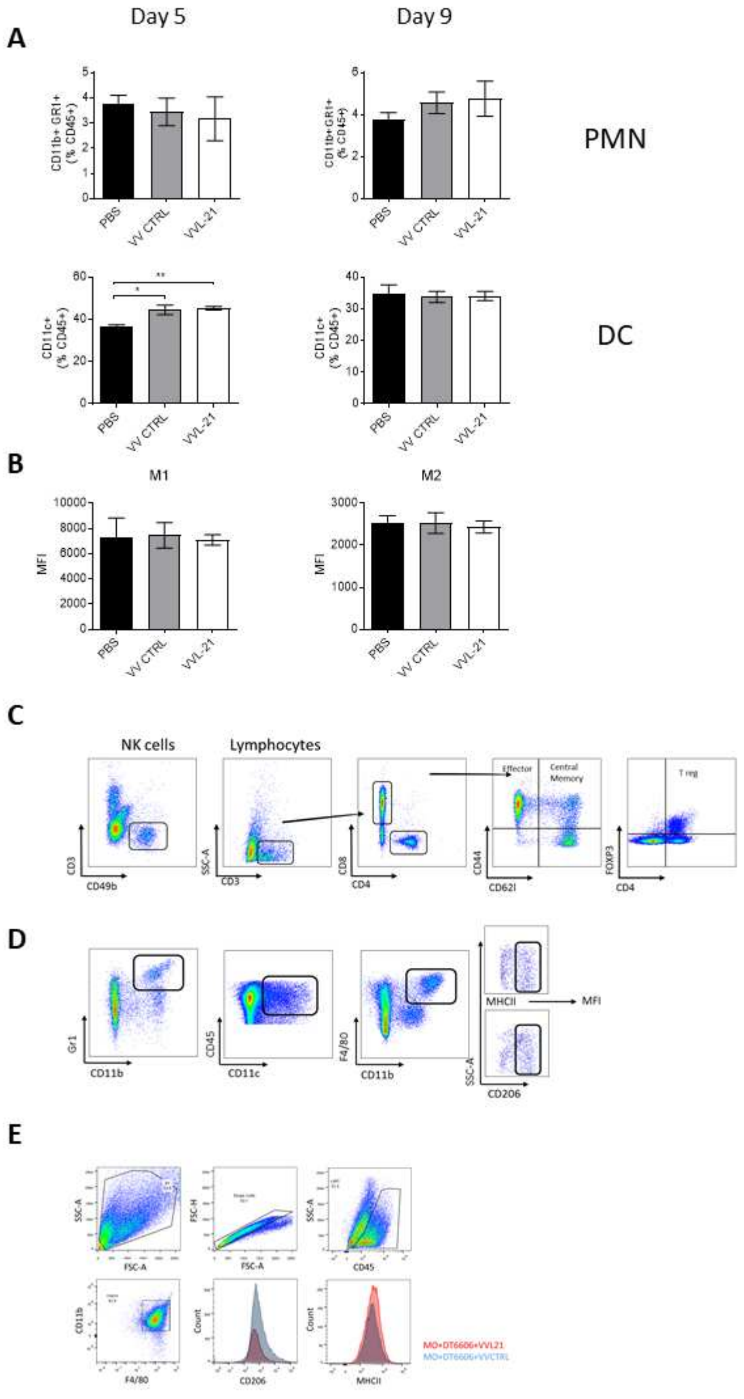

Supplementary figure 5. VVL-21 treatment does not alter the PMN or DC populations within the tumor. DT6606 tumors were established subcutaneously in immunocompetent C57Bl/6 mice. Once palpable, mice were treated with CAL-101 (10mg/Kg) by oral gavage followed 3 hours later by i.v injection using 1x10^8 PFU/injection. 1 or 5 days post last treatment (day 5 or day 9), tumors were analyzed for the presence of PMN or DC (**A**) using flow cytometry. Mean percentage of cells +/- SEM is shown and a one-way ANOVA with Tukey's multiple comparison post-test was used to determine significance. n=3/group. (**B**) An assessment of intra-tumoral macrophage polarization at day 6 is shown. MFI of MHCII is shown and a one-way ANOVA with Tukey's multiple comparison post-test was used to determine significance. n=3/group. (**C** and **D**) The FACS gating strategy for adaptive (**C**) and innate (**D**) immune populations is shown. (**E**) The gating strategy for M1 and M2 macrophages is shown in detail. *p<0.05; ** p<0.01.

Marelli G, et al. J Immunother Cancer 2021; 9:e001624. doi: 10.1136/jitc-2020-001624

Supplementary figure 6

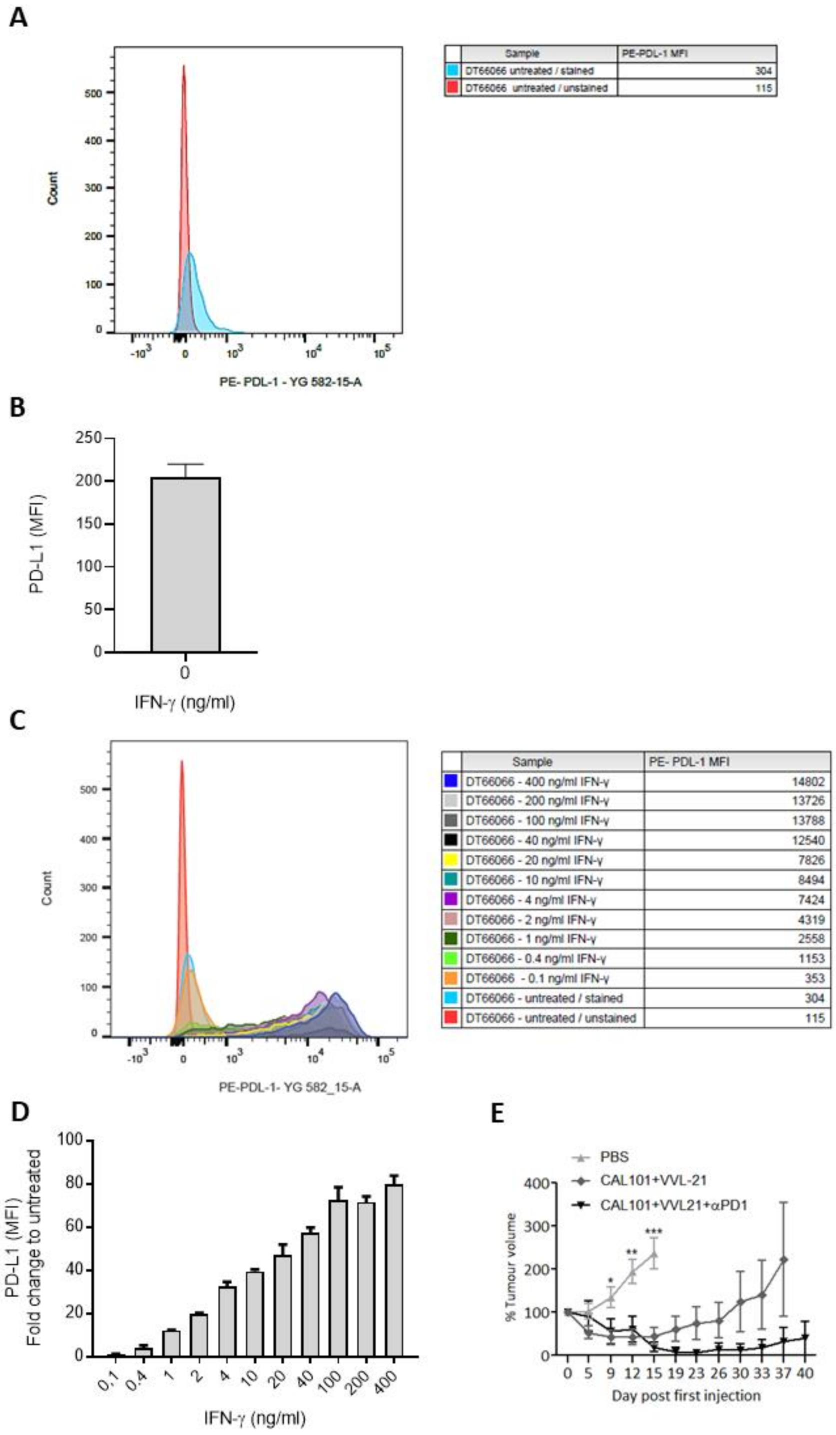

Supplementary figure 6. DT6606 tumor cells up-regulate PD-L1 expression in response to IFN-γ treatment *in vitro*. DT6606 cells were seeded in 6 well plates and treated with varying concentrations of murine IFN-γ as indicated for 48 hours. Cells were collected and analyzed using flow cytometry to assess PD-L1 expression. (**A** and **B**) MFI of cells stained with PE-conjugated antibody recognising PD-L1 was compared to unstained sample. (**C** and **D**) The effect of IFN-γ on PD-L1 expression was assessed by incubating DT6606 cells with 11 different concentrations of recombinant protein. PE-MFI was compared to untreated sample (**C**) and plotted as fold-changed (**D**) showing an increase in the intensity of the signal with the increase of IFN-γ concentration. n=3/group. (**E**) DT6606 tumors were established subcutaneously in immunocompetent C57Bl/6 mice. Once palpable, mice were treated with CAL-101 (10mg/Kg) by oral gavage followed 3 hours later by i.v injection using $1x10^8$ PFU/injection on days 0, 2 and 4. α-PD1 was administered on days 2, 5 and 7. Tumor size was monitored twice weekly and the mean +/- SEM is shown. Significance was assessed using a two-way ANOVA with Tukey's multiple comparison post-test and comparison between PBS and both treatment groups is shown until day 15. n=5-7/group. *p<0.05; ** p<0.01; *** p<0.001.

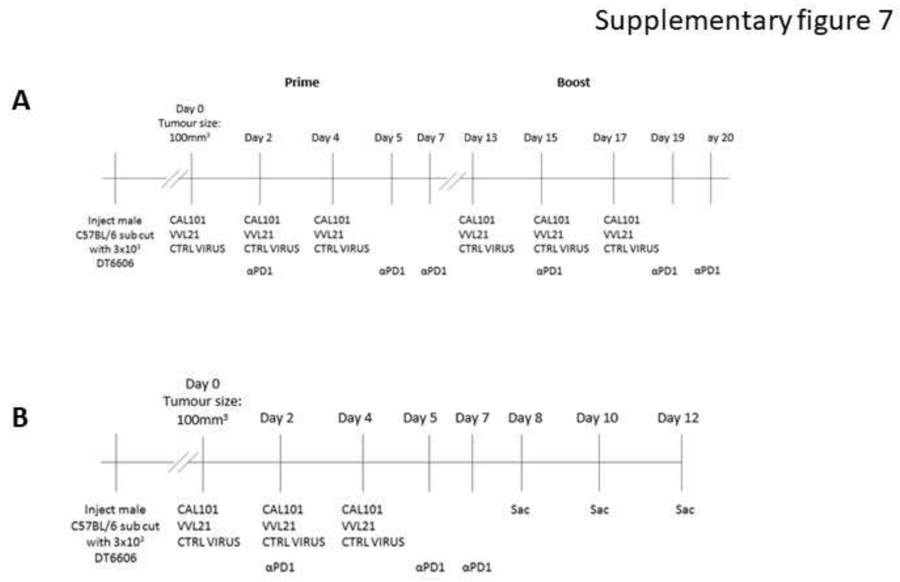

Supplementary figure 7. Schematic for *in vivo* treatment protocols. DT6606 tumors were established in immunocompetent C57Bl/6 mice. (**A**) Once tumors reached a volume of 100mm^3 they were treated for efficacy studies as shown, with injections of virus at a PFU of 1x10^8 for injections on days 0, 2, 4 and 2x10^8 on days 13, 15, 17. α-PD1 was administered by i.p injection on days 2, 5, 7, 15, 19 and 20 (200μg/injection). (**B**) Once tumors reached a volume of 100mm^3 they were treated for functional studies as shown, with injections of virus at a PFU of 1x10^8 for injections on days 0, 2, 4. α-PD1 was administered by i.p injection on days 2, 5 and 7 (200μg/injection).
